# Activation of the adhesion G protein–coupled receptor GPR133 by antibodies targeting its N-terminus

**DOI:** 10.1016/j.jbc.2022.101949

**Published:** 2022-04-18

**Authors:** Gabriele Stephan, Joshua D. Frenster, Ines Liebscher, Dimitris G. Placantonakis

**Affiliations:** 1 Department of Neurosurgery, NYU Grossman School of Medicine, New York, New York, USA; 2 Rudolf Schönheimer Institute for Biochemistry, Molecular Biochemistry, University of Leipzig, Leipzig, Germany; 3 Kimmel Center for Stem Cell Biology, NYU Grossman School of Medicine, New York, New York, USA; 4 Laura and Isaac Perlmutter Cancer Center, NYU Grossman School of Medicine, New York, New York, USA; 5 Brain and Spine Tumor Center, NYU Grossman School of Medicine, New York, New York, USA; 6 Neuroscience Institute, NYU Grossman School of Medicine, New York, New York, USA

**Keywords:** adhesion G protein-coupled receptor, antibody, signaling, dissociation, aGPCRs, adhesion G protein-coupled receptors, BSA, bovine serum albumin, CTF, C-terminal fragment, FBS, fetal bovine serum, GAIN, GPCR autoproteolysis-inducing, GBM, glioblastoma, GPS, GPCR proteolysis site, HA, hemagglutinin, HEK293T, Human embryonic kidney 293 T, HTRF, homogeneous time resolved fluorescence, IBMX, 3-isobutyl-1-methylxanthine, NTF, N-terminal fragment, pCTRL, control peptide, PTX, pentraxin domain

## Abstract

We recently demonstrated that GPR133 (ADGRD1), an adhesion G protein–coupled receptor involved in raising cytosolic cAMP levels, is necessary for growth of glioblastoma (GBM) and is *de novo* expressed in GBM relative to normal brain tissue. Our previous work suggested that dissociation of autoproteolytically generated N-terminal and C-terminal fragments of GPR133 at the plasma membrane correlates with receptor activation and signaling. To promote the goal of developing biologics that modulate GPR133 function, we investigated the effects of antibodies against the N-terminus of GPR133 on receptor signaling. Here, we show that treatment of HEK293T cells overexpressing GPR133 with these antibodies increased cAMP levels in a concentration-dependent manner. Analysis of culture medium following antibody treatment further indicated the presence of complexes of these antibodies with the autoproteolytically cleaved N-terminal fragments of GPR133. In addition, cells expressing a cleavage-deficient mutant of GPR133 (H543R) did not respond to antibody stimulation, suggesting that the effect is cleavage dependent. Finally, we demonstrate the antibody-mediated stimulation of WT GPR133, but not the cleavage-deficient H543R mutant, was reproducible in patient-derived GBM cells. These findings provide a paradigm for modulation of GPR133 function with biologics and support the hypothesis that the intramolecular cleavage in the N-terminus modulates receptor activation and signaling.

Adhesion G protein–coupled receptors (aGPCRs) represent the second largest subfamily within the GPCR superfamily (1, 2) and have been implicated in numerous physiological processes and disease mechanisms (3, 4, 5). Adhesion GPCRs are structurally characterized by an intracellular C-terminus, a seven transmembrane segment domain and a large extracellular N-terminus (2, 6, 7). While distinct functional domains within the N-terminus are thought to mediate receptor-specific interactions with adjacent cells or the extracellular matrix (2), almost all aGPCRs share a conserved GPCR autoproteolysis-inducing (GAIN) domain within the N-terminus. This domain catalyzes intramolecular autoproteolytic cleavage at the GPCR proteolysis site (GPS) within the N-terminus, resulting in an N-terminal fragment (NTF) and a C-terminal fragment (CTF) (8). A prevalent hypothesis in the field is that binding of ligands from adjacent cells or the extracellular matrix to the N-terminus, as well as mechanical stimuli, induce conformational changes or NTF-CTF dissociation (3, 9, 10, 11, 12). These events, in turn, enable the *Stachel* sequence (9, 13, 14, 15, 16, 17, 18, 19), a tethered internal agonist peptide sequence immediately distal to the GPS, to activate signaling (3, 20). However, the exact activation mechanisms likely differ among members of the aGPCR family and are not well characterized.

Our group recently demonstrated part of the mechanism that mediates the activation of GPR133 (ADGRD1), a member of group V of aGPCRs (2) implicated in the pathogenesis of glioblastoma (GBM) (21, 22), an aggressive brain malignancy (23). The N-terminus of GPR133, which contains a pentraxin (PTX) domain, undergoes autoproteolytic cleavage almost immediately after protein synthesis (24). However, NTF and CTF stay noncovalently bound to each other until they are trafficked to the plasma membrane, where their dissociation occurs and correlates with increased signaling mediated by G_αs_, resulting in activation of adenylate cyclase and elevation in cAMP levels (21, 24, 25, 26, 27). Our finding that dissociation of NTF and CTF correlates with increased signaling is in accordance with the previous observation that the CTF of GPR133, when expressed without the NTF, demonstrates hyperactive signaling relative to its full-length counterpart (9). Collectively, our data suggest that the cleaved but noncovalently associated NTF-CTF holoreceptor is signaling competent, but its dissociation at the plasma membrane enables full activation of receptor signaling.

Here, we demonstrate that antibodies targeting epitopes outside of the GAIN domain of the N-terminus of GPR133 increase receptor-mediated G_αs_ signaling and cAMP levels. Preventing specific antibody binding by deleting the targeted epitope abolishes the effect. The antibody-mediated activation is dependent on receptor cleavage, because antibodies fail to modulate signaling of a cleavage-deficient GPR133 mutant (H543R). These findings suggest that GPR133 function can be modulated by antibodies, and likely other biologics as well, which can be used as molecular tools in the study of receptor activation but also as therapeutic platforms in the context of GBM and possibly other malignancies, where GPR133 plays important roles.

## Results

### Activation of GPR133 signaling with antibodies against its N-terminus

**Figure 1:**
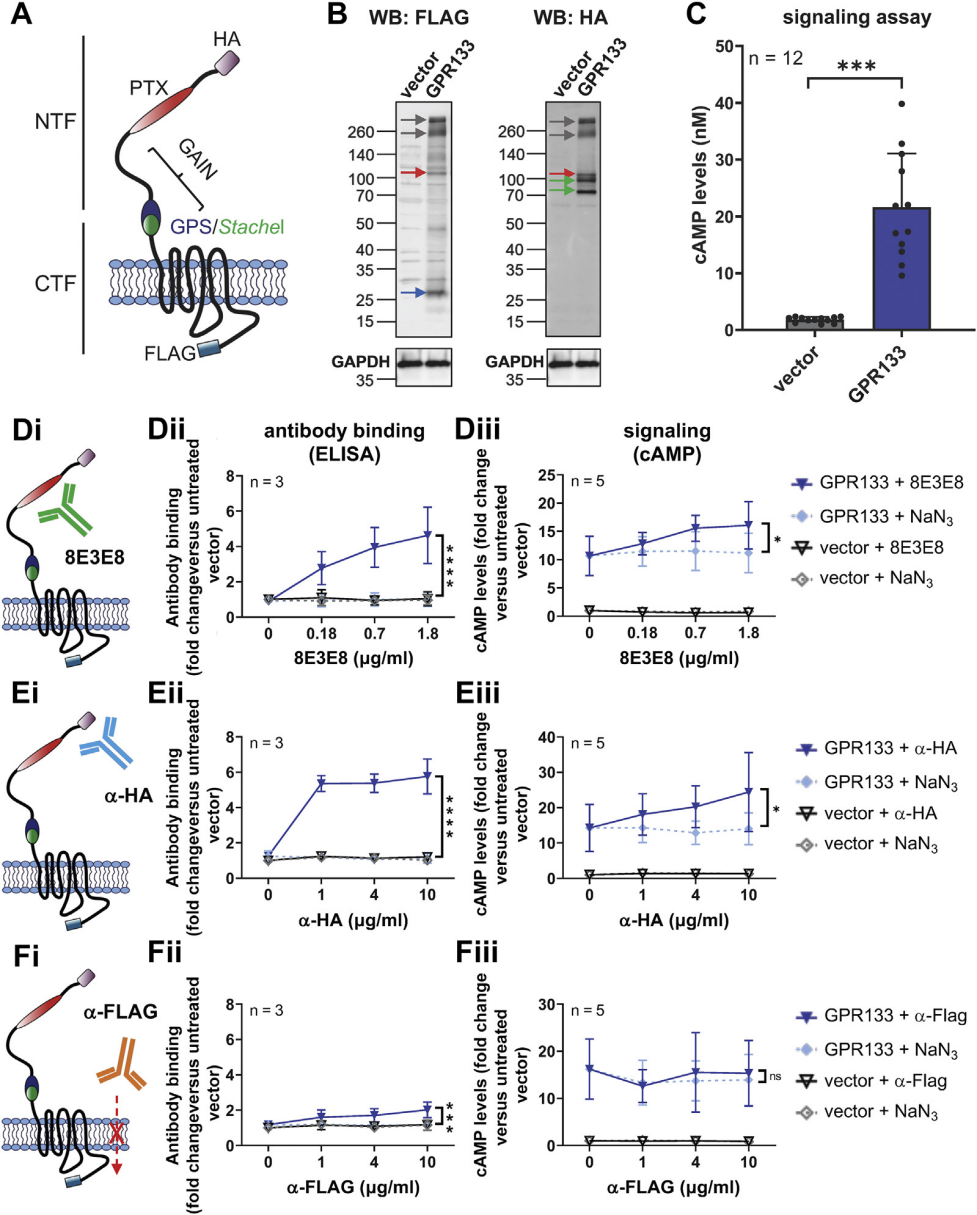
**Antibody stimulation increases cAMP levels in HEK293T cells overexpressing GPR133.***A*, a double-tagged GPR133 construct, including an N-terminal HA-tag and a C-terminal FLAG-tag, was used in these experiments. *B*, overexpression of GPR133 is shown by Western blots of whole cell lysates. The same membrane was simultaneously probed with an anti-HA antibody (α-HA; targeting the N-terminal HA-tag), an anti-FLAG antibody (α-FLAG; targeting the C-terminal FLAG-tag), and an anti-GAPDH antibody. The GAPDH image was reused for single images of the α-HA and anti-FLAG blots. The full-length receptor (*red arrow*), the CTF (*blue arrow*), and the NTF (*green arrow*) are detected in cells transfected with GPR133. *C*, cAMP levels significantly increase after overexpression of GPR133. Bars represent mean ± SD of four individual experiments, ∗∗∗*p* < 0.001, *t* test. *D*–*F*, antibody binding, assessed by ELISA and cAMP levels following the treatment of HEK293T cells overexpressing GPR133 with different antibodies. NaN_3_ (0.015 mM, 0.06 mM, 0.15 mM) served as a solvent control. Data points represent mean ± SD of 3 to 5 individual experiments. The GPR133 + NaN_3_ and GPR133 + 8E3E8 (or α-HA, or α-FLAG) groups were compared by two-way ANOVA (∗*p* < 0.05; ∗∗∗*p* < 0.001; ∗∗∗∗*p* < 0.0001). *Di*, 8E3E8 targets the N-terminal PTX domain, and (*Dii*) binds to GPR133 in a dose-dependent manner in ELISA assays (F_(1,16)_ = 41.31, *p* < 0.0001). *Diii*, treatment with 8E3E8 leads to a concentration-dependent increase of cAMP levels compared to the NaN_3_ treatment when overexpressing GPR133 (F_(1,32)_ = 6.509, *p* = 0.0157; untreated vector = 1.79 ± 0.25 nM cAMP). *Ei*, the α-HA antibody targets an N-terminal HA tag and (*Eii*) shows binding to GPR133-expressing cells on ELISA assays (F_(1,16)_ = 284.6, *p* < 0.0001). *Eiii*, treatment with α-HA antibody significantly increases cAMP levels compared to the NaN_3_ control (F_(1,32)_= 6.997; *p* = 0.0125; untreated vector = 1.61 ± 0.25 nM cAMP). *Fi*, HEK293T cells overexpressing GPR133 were treated with α-FLAG antibody targeting the intracellular C-terminus. *Fii*, Binding of α-FLAG to GPR133 was statistically significant (F_(1,16)_ = 17.09, *p* = 0.0008) but much less prominent than binding of 8E3E8 or α-HA, and in (*Fiii*), there were no significant changes in cAMP concentrations (F_(1,32)_ = 0.1115, *p* = 0.7407, two-way ANOVA; untreated vector = 1.57 ± 0.24 nM cAMP). CTF, C-terminal fragment; HA, hemagglutinin; HEK293T, human embryonic kidney 293T; NTF, N-terminal fragment; PTX, pentraxin domain.

To test whether GPR133 signaling is modulated by antibodies binding to the extracellular N-terminus, we transfected Human embryonic kidney 293T (HEK293T) cells with GPR133 tagged with an N-terminal hemagglutinin (HA) and a C-terminal FLAG epitope (Fig. 1*A*). Overexpression of tagged GPR133 was verified by Western blot analysis of whole cell lysates 48 h after transfection (Fig. 1*B*). As expected (9, 24, 27), staining with an anti-FLAG antibody detected the CTF (blue arrow, ∼25 kDa), staining with an anti-HA antibody detected bands representing the maturely and immaturely glycosylated NTF (green arrows, ∼95/75 kDa), and both antibodies detected small amounts of the full-length uncleaved receptor (red arrows, ∼110 kDa). The band sizes of the full-length receptor, detected with the anti-FLAG and anti-HA antibodies, and the NTF, as detected by the anti-HA antibody, as well as the shift from the expected molecular weight of the CTF (∼36 kDa) to the observed molecular weight (blue arrow, ∼25 kDa), are in agreement with our previous findings (24). Indeed, we previously showed that glycosylation increases the apparent molecular weight of the NTF (24). The size shift of the CTF is most likely caused by increased loading of SDS to hydrophobic transmembrane regions of the CTF (28). Moreover, both antibodies detected bands >260 kDa (gray arrows), presumably representing aggregates of the receptor.

We used a homogeneous time resolved fluorescence (HTRF)-based assay to quantify cAMP concentrations after expression of GPR133 (Fig. 1*C*). In agreement with previously published data (24, 25, 27), intracellular cAMP levels increased significantly in HEK293T cells overexpressing GPR133 relative to cells transfected with the empty vector (*p* < 0.001, *t* test).

We then treated the HEK293T cells with either a mouse monoclonal antibody (8E3E8) that we raised against the PTX domain of GPR133 (Fig. 1*D*i) (21, 29), a commercial anti-HA antibody (Fig. 1*E*i), or a commercial anti-FLAG antibody (Fig. 1*F*i). A range of antibody concentrations was tested. Since these antibodies were stored in solution containing NaN_3_, increasing concentrations of NaN_3_ (0.015 mM, 0.06 mM, 0.15 mM) served as a control. To verify binding of the antibodies to GPR133, we performed an ELISA under nonpermeabilizing conditions (Fig. 1, *D*ii, *E*ii, *F*ii). Optical density increased proportionally with increasing concentrations of the extracellular antibodies (8E3E8: F _(1,16)_ = 41.31, *p* < 0.0001; anti-HA: F _(1,16)_ = 284.6, *p* < 0.0001; two-way ANOVA) compared to the NaN_3_ control. The anti-FLAG antibody, which recognizes the intracellular FLAG epitope, also showed a slight concentration-dependent increase (F _(1,16)_ = 17.09, *p* = 0.0008; two-way ANOVA). However, this ELISA signal was dramatically smaller than with the 8E3E8 and anti-HA antibodies. The increase in optical density after applying the FLAG antibody might be due to partial permeabilization of the plasma membrane during fixation of the cells.

**Supplementary Figure 1:**
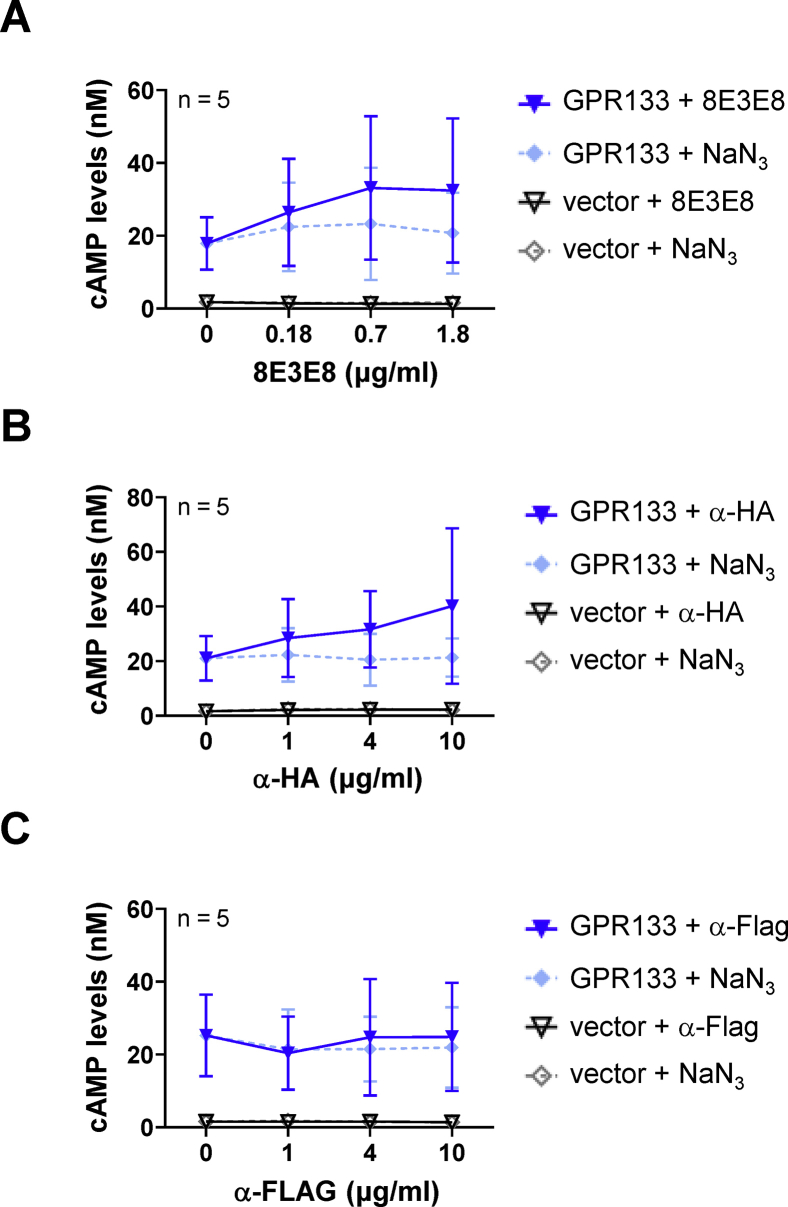


To test the effect of antibodies on GPR133 signaling, we quantified intracellular cAMP levels following stimulation of HEK293T cells transfected with the empty vector or GPR133. We found significant concentration-dependent increases in cAMP following the treatment with 8E3E8 (F _(1,32)_ = 6.509, *p* = 0.0157, two-way ANOVA) and anti-HA antibodies (F _(1,32)_ = 6.997, *p* = 0.0125, two-way ANOVA), but not the anti-FLAG antibody (F _(1,32)_ = 0.1115, *p* = 0.7407, two-way ANOVA) (Fig. 1, *D*iii, *E*iii, *F*iii; raw cAMP levels in nM are shown in Fig. S1). We observed a 1.4-fold increase in cAMP levels following treatment with 1.8 μg/ml 8E3E8 (fold change in cAMP levels relative to the untreated empty vector condition: GPR133 + NaN_3_ = 11.2 ± 1.5 *versus* GPR133 + 8E3E8 = 16.1 ± 1.9) and a 1.7-fold increase following treatment with 10 μg/ml anti-HA (fold change in cAMP levels relative to untreated empty vector condition: GPR133 + NaN_3_ = 14.0 ± 2.0 *versus* GPR133 + anti-HA = 24.4 ± 4.9). Signaling did not increase following treatment with 10 μg/ml anti-FLAG (fold change in cAMP levels relative to untreated empty vector condition: GPR133 + NaN_3_ = 13.9 ± 2.4 *versus* GPR133 + anti-FLAG = 15.3 ± 3.1). These findings suggest that antibodies targeting the NTF of GPR133 outside of the GAIN domain increase receptor signaling in HEK293T cells.

**Figure 2:**
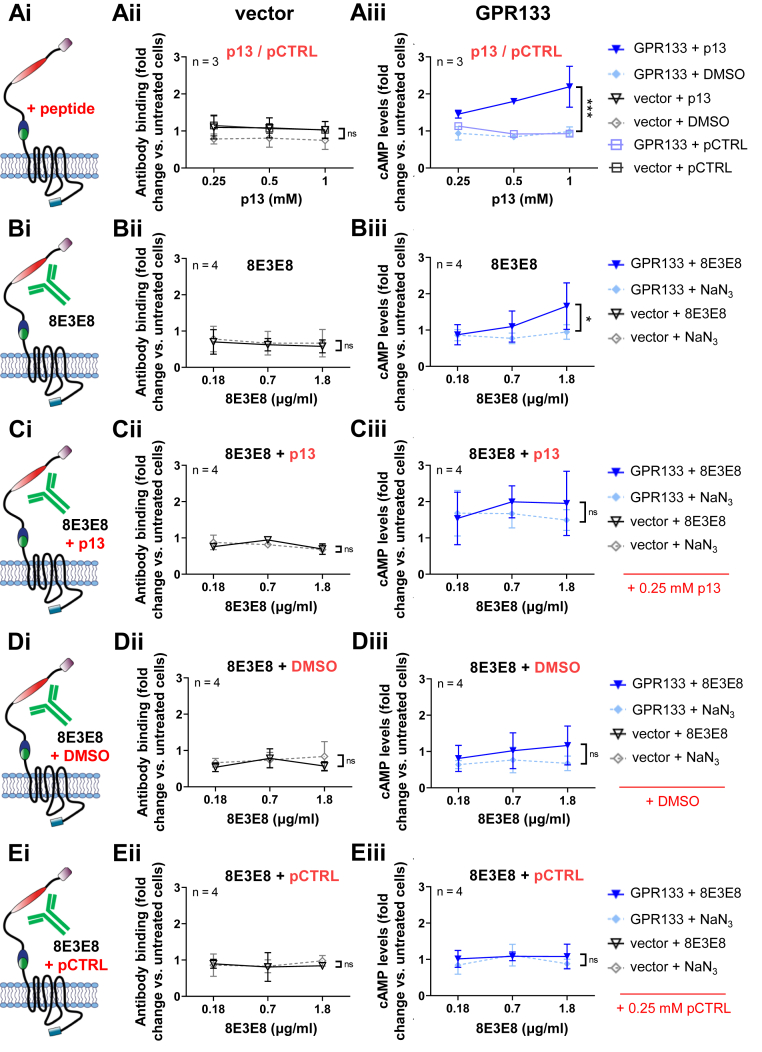
**Effects of 8E3E8 and the *Stachel*-derived peptide on GPR133 signaling.** HEK293T cells overexpressing the empty vector or HA- and Flag-tagged GPR133 were treated with different concentrations of (*A*) the *Stachel*-derived peptide p13; or (*B*) 8E3E8. NaN_3_ (0.015 mM, 0.06 mM, 0.15 mM) served as a solvent control. *A*, treatment with increasing concentrations of p13 resulted in increased cAMP levels compared to the DMSO treatment in GPR133-expressing cells (F_(1,12)_ = 19.18, ∗∗∗*p* = 0.0009). *B*, treatment with 8E3E8 led to a concentration-dependent increase of cAMP levels compared to the NaN_3_ treatment in GPR133-expressing cells (F_(1,18)_ = 5.862, ∗*p* = 0.0263). *C*–*E*, cotreatment with 8E3E8 and either (C) p13, (D) DMSO, or (E) pCTRL. *C*, costimulation with 0.25 mM p13 resulted in an increased baseline of GPR133 signaling across all concentrations of 8E3E8, but p13 prevented the concentration-dependent increases in cAMP. *D* and *E*, treatment with 8E3E8 and 0.25% DMSO (D) or 0.25 mM pCTRL (*E*) did not increase cAMP levels compared to untreated cells. Data on the y-axis represent cAMP levels normalized to the untreated cells. Data points represent mean ± SD of four individual experiments. The GPR133 + DMSO and GPR133 + p13/pCTRL groups or the GPR133 + NaN3 and GPR133 + 8E3E8 groups were compared by two-way ANOVA. HA, hemagglutinin; HEK293T, human embryonic kidney 293T; pCTRL, control peptide.

**Supplementary Figure 2:**
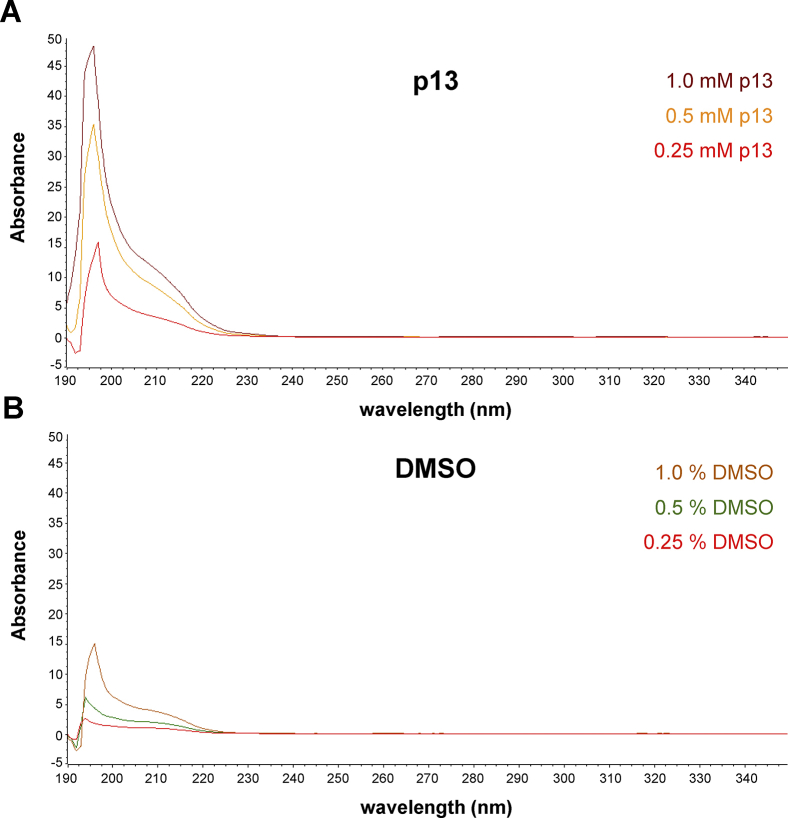


**Supplementary Figure 3:**
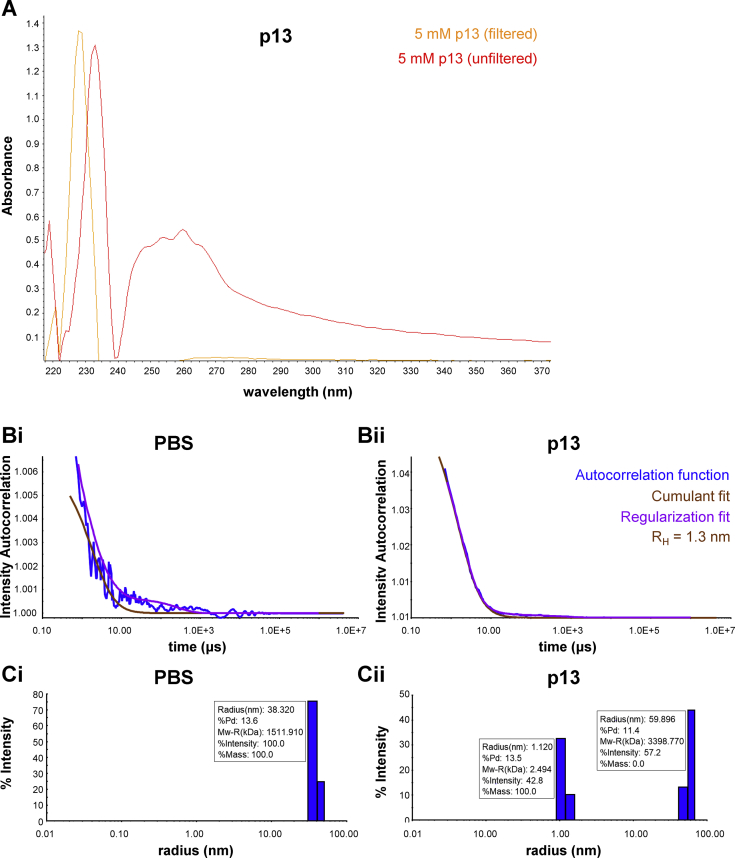


**Supplementary Figure 4:**
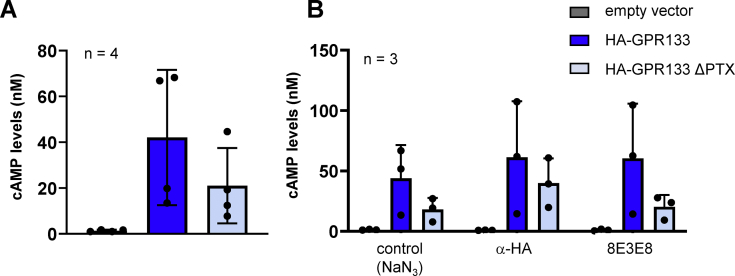


Next, we compared the effect of the antibodies on HEK293T cells overexpressing GPR133 with the receptor’s natural activation mechanism, by using a *Stachel*-derived soluble peptide (9) and DMSO-containing medium as a control (Fig. 2; raw cAMP levels in nM are shown in Fig. S2). The soluble 13 amino acid-long peptide (p13) mimics the endogenous agonistic *Stachel* sequence, thereby specifically activating GPR133 (9). Solubility of the *Stachel*-derived peptide in aqueous solution was confirmed by measuring the absorbance of peptide solutions in different concentrations (Fig. S3). Indeed, peak absorbance at 195 nm increased in concentration-dependent fashion with increasing concentrations of p13, suggesting p13 is soluble at the concentrations used in our experiments. Dynamic light scattering measurements further validated the solubility of p13 (Fig. S4). As described previously (9), treatment with p13 resulted in a concentration-dependent increase of GPR133 signaling (F _(1,12)_ = 19.8, *p* = 0.0009, two-way ANOVA) (Fig. 2*A*). We observed a 1.5-fold increase in cAMP levels following treatment with a submaximal concentration of p13 (0.25 mM) relative to DMSO, which is comparable to the magnitude of increase following antibody stimulation. We then stimulated GPR133 with increasing concentrations of 8E3E8 alone (Fig. 2*B*) or in combination with 0.25 mM p13 (Fig. 2*C*). Different concentrations of NaN_3_ were used as a control. Treatment with 0.25 mM p13 increased the baseline of the 8E3E8 (or NaN_3_) concentration-response curve (Fig. 2*C*) and blunted the dose-dependent increase in cAMP with increasing 8E3E8 concentrations (F _(1,18)_ = 0.77, *p* = 0.3916, two way ANOVA). In contrast, treatment with both 8E3E8 and DMSO vehicle impaired the 8E3E8-induced increase in cAMP (F _(1,18)_ = 4.0, *p* = 0.0597, two way ANOVA) (Fig. 2*D*), suggesting nonspecific effects of DMSO on antibody–GPR133 interactions. Similarly, an inactive control peptide (pCTRL), which does not increase cAMP production by GPR133 (F _(1,18)_ = 1.7, *p* = 0.2113, two way ANOVA) (Fig. 2*A*), blocked the effects of 8E3E8 (F _(1,18)_ = 1.3, *p* = 0.2728, two way ANOVA) (Fig. 2*E*). Collectively, these results indicate that cotreatment with a submaximal concentration of p13 blunts the dose-dependent agonistic effects of 8E3E8, which suggests that the antibody-induced increase in cAMP may be mediated by the endogenous *Stachel* agonist sequence.

**Figure 3:**
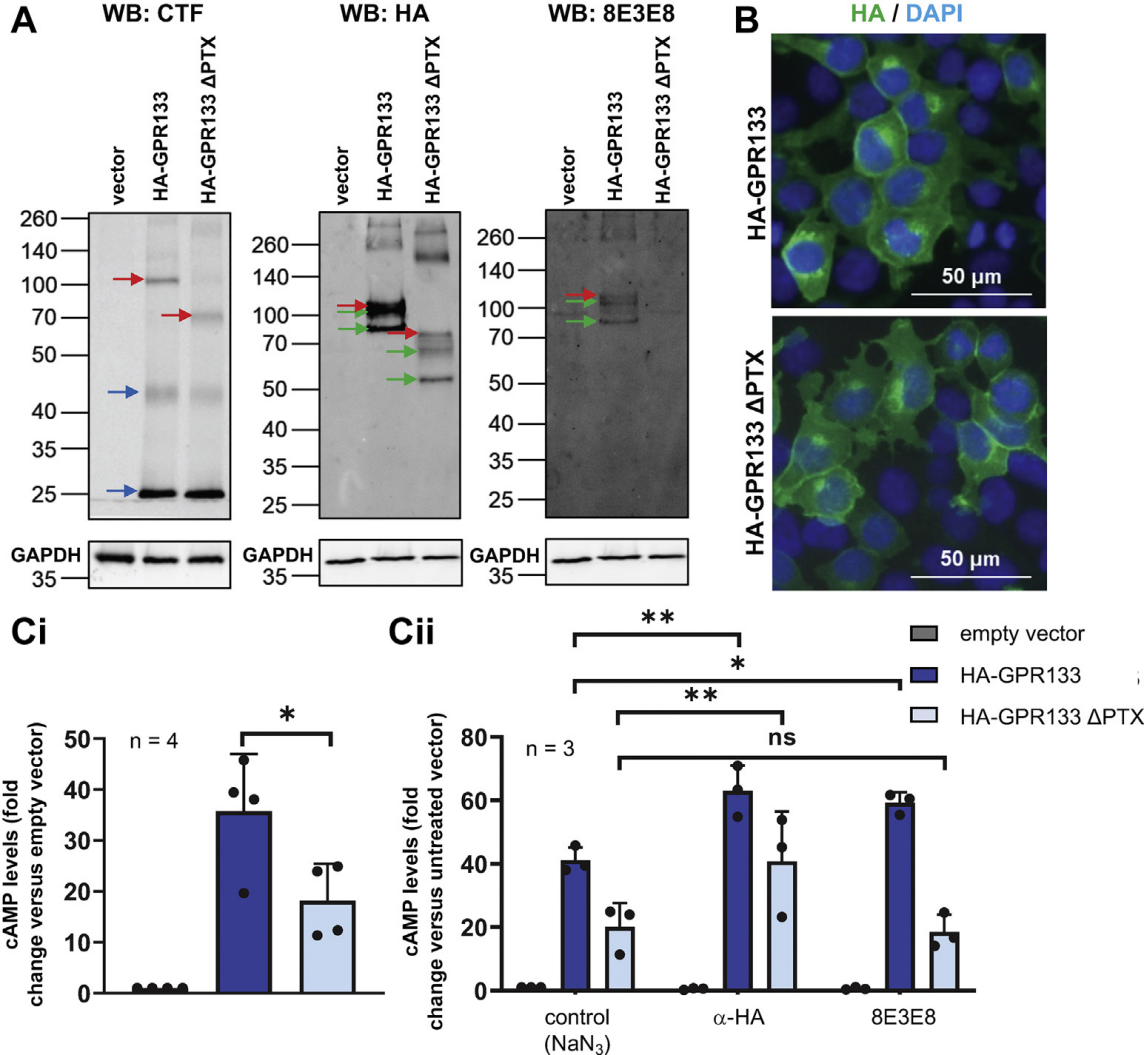
**Specific binding of the antibody to the GPR133 PTX domain activates the receptor.***A*, Western blot analysis of whole cell lysates of HEK293T cells overexpressing HA-GPR133 and the deletion mutant HA-GPR133 ΔPTX compared to an empty vector. Detection with α-HA and anti-CTF antibody shows the full-length receptor (*red arrow*), the NTF (*green arrow*), and the CTF (*blue arrow*). Size shifts of the full-length receptor and the NTF are related to the PTX deletion. 8E3E8 detects HA-GPR133, but not the deletion mutant. The same membrane was stained with α-HA, 8E3E8, and anti-GAPDH antibodies. The GAPDH image was reused for single images of the α-HA and 8E3E8 blots. *B*, immunostaining of HEK293T cells overexpressing HA-GPR133 and HA-GPR133 ΔPTX with α-HA antibody (*green*) and DAPI (*blue*) shows that both constructs can be detected at the plasma membrane. *C*, cAMP responses of HEK293T cells overexpressing HA-GPR133 and HA-GPR133 ΔPTX. The bars represent mean ± SD of 3 to 4 individual experiments (∗*p* < 0.05; ∗∗*p* < 0.01). *Ci*, deletion of the PTX domain significantly reduces cAMP levels (F_(2,9)_ = 20.24; *p* = 0.0005, one-way ANOVA; Tukey’s *post hoc* test for HA-GPR133 compared to HA-GPR133 ΔPTX, ∗*p* = 0.0256). Data on the y-axis represent cAMP levels normalized to the untreated vector condition (untreated vector = 1.33 ± 0.38 nM). *Cii*, HA-GPR133 signaling is increased significantly by adding α-HA antibody, binding the N-terminal HA-tag, or 8E3E8, binding the PTX domain. In contrast, HA-GPR133 ΔPTX signaling is increased only by α-HA, but not 8E3E8. (F_(2,18)_ = 9.490; *p* = 0.0015, two-way ANOVA; Tukey’s *post hoc* test: HA-GPR133 control *versus* HA-GPR133 + α-HA, *p* = 0.0029; HA-GPR133 control *versus* HA-GPR133 + 8E3E8, *p* = 0.0126; HA-GPR133 ΔPTX control *versus* HA-GPR133 ΔPTX + α-HA, *p* = 0.0047; HA-GPR133 ΔPTX control *versus* HA-GPR133 ΔPTX + 8E3E8, *p* = 0.9558). Data on the y-axis represent cAMP levels normalized to the untreated vector condition (untreated vector = 1.28 ± 0.36 nM). CTF, C-terminal fragment; HA, hemagglutinin; HEK293T, human embryonic kidney 293T; NTF, N-terminal fragment; PTX, pentraxin domain.

**Supplementary Figure 5:**
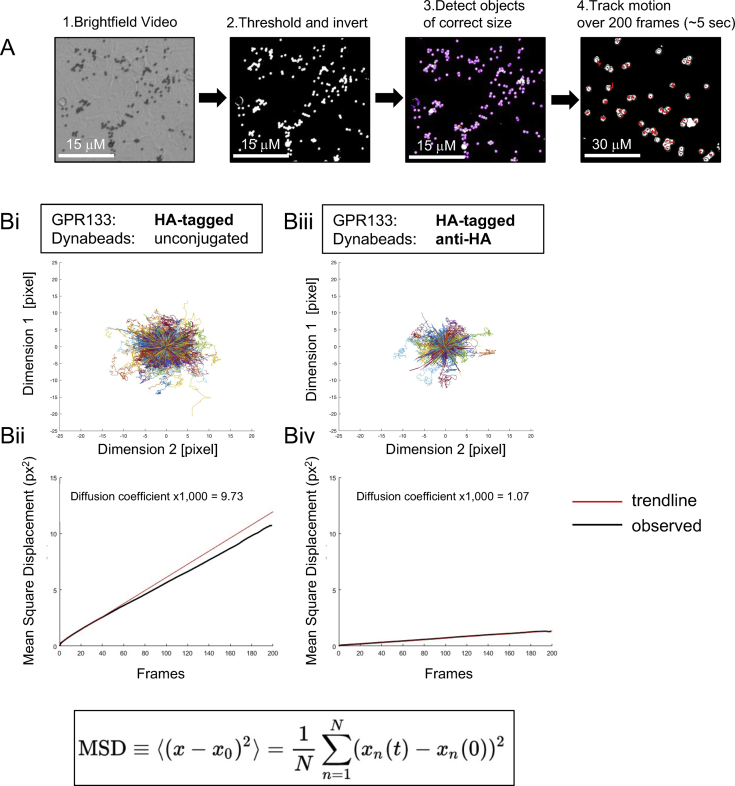


To ascertain the specificity of the activating effect of 8E3E8, we deleted the PTX domain (amino acids 79–276) of GPR133, which contains the epitope that 8E3E8 recognizes. The deletion is predicted to cause a 22 kDa decrease in molecular weight. Overexpression of HA-tagged GPR133 with the PTX deletion (HA-GPR133 ΔPTX) was confirmed by Western blot analysis of whole cell lysates (Fig. 3*A*). Staining with the PTX-recognizing 8E3E8 antibody detected HA-GPR133 but not HA-GPR133 ΔPTX, confirming the PTX deletion (Fig. 3*A*). Staining with the anti-HA antibody and a commercial antibody against the cytosolic C-terminus of GPR133 (anti-CTF) demonstrated the expected size shifts of the full-length receptor and the NTF after deletion of the PTX domain (Fig. 3*A*). Importantly, deletion of the PTX domain did not impair receptor cleavage. Immunofluorescent staining of HEK293T cells overexpressing either full-length HA-GPR133 or HA-GPR133 ΔPTX with an anti-HA antibody showed similar staining patterns, suggesting the subcellular localization and membrane trafficking of the mutant receptor is not altered (Fig. 3*B*). The baseline levels of cAMP were significantly reduced in HEK293T cells overexpressing HA-GPR133 ΔPTX compared to cells overexpressing HA-GPR133 (F _(2,9)_ = 20.24, *p* = 0.0005, one-way ANOVA; Tukey’s *post hoc* test: HA-GPR133 compared to HA-GPR133 ΔPTX, *p* = 0.0256) (Fig. 3*C*i; raw cAMP levels in nM are shown in Fig. S5*A*). Of note, basal cAMP levels of HA-GPR133 are higher than the basal activity of GPR133 tagged with both the N-terminal HA and the C-terminal FLAG epitopes (Fig. 1), because we found that the C-terminal FLAG tag mildly reduces GPR133 signaling (data not shown). While treatment of cells overexpressing HA-GPR133 with either 10 μg/ml anti-HA or 1.8 μg/ml 8E3E8 activated receptor signaling equivalently (anti-HA = 1.5-fold, fold change in cAMP levels relative to untreated empty vector condition: HA-GPR133 + NaN_3_ = 41.1 ± 4.1 *versus* HA-GPR133 + anti-HA = 63.0 ± 8.0; 8E3E8 = 1.4-fold, fold change in cAMP levels relative to untreated empty vector condition: HA-GPR133 + NaN_3_ = 41.1 ± 4.1 *versus* HA-GPR133 + 8E3E8 = 59.2 ± 3.3), only treatment with anti-HA (2-fold, fold change in cAMP levels relative to untreated empty vector condition: HA-GPR133 ΔPTX + NaN_3_ = 20.1 ± 7.6 *versus* HA-GPR133 ΔPTX + anti-HA = 40.7 ± 15.8) but not 8E3E8 (fold change in cAMP levels relative to untreated empty vector condition: HA-GPR133 ΔPTX + NaN_3_ = 20.1 ± 7.6 *versus* HA-GPR133 ΔPTX + 8E3E8 = 18.5 ± 5.5) increased cAMP levels in cells expressing HA-GPR133 ΔPTX (F _(2,18)_ = 9.490, *p* = 0.0015, two-way ANOVA; Tukey’s *post hoc* test: HA-GPR133 control *versus* HA-GPR133 + α-HA, *p* = 0.0029; HA-GPR133 control *versus* HA-GPR133 + 8E3E8, *p* = 0.0126; HA-GPR133 ΔPTX control *versus* HA-GPR133 ΔPTX + α-HA, *p* = 0.0047; HA-GPR133 ΔPTX control *versus* HA-GPR133 ΔPTX + 8E3E8, *p* = 0.9558) (Fig. 3*C*ii; raw cAMP levels in nM are shown in Fig. S5*B*). This finding indicated specificity of the activating effect of 8E3E8 on GPR133 signaling.

**Figure 4:**
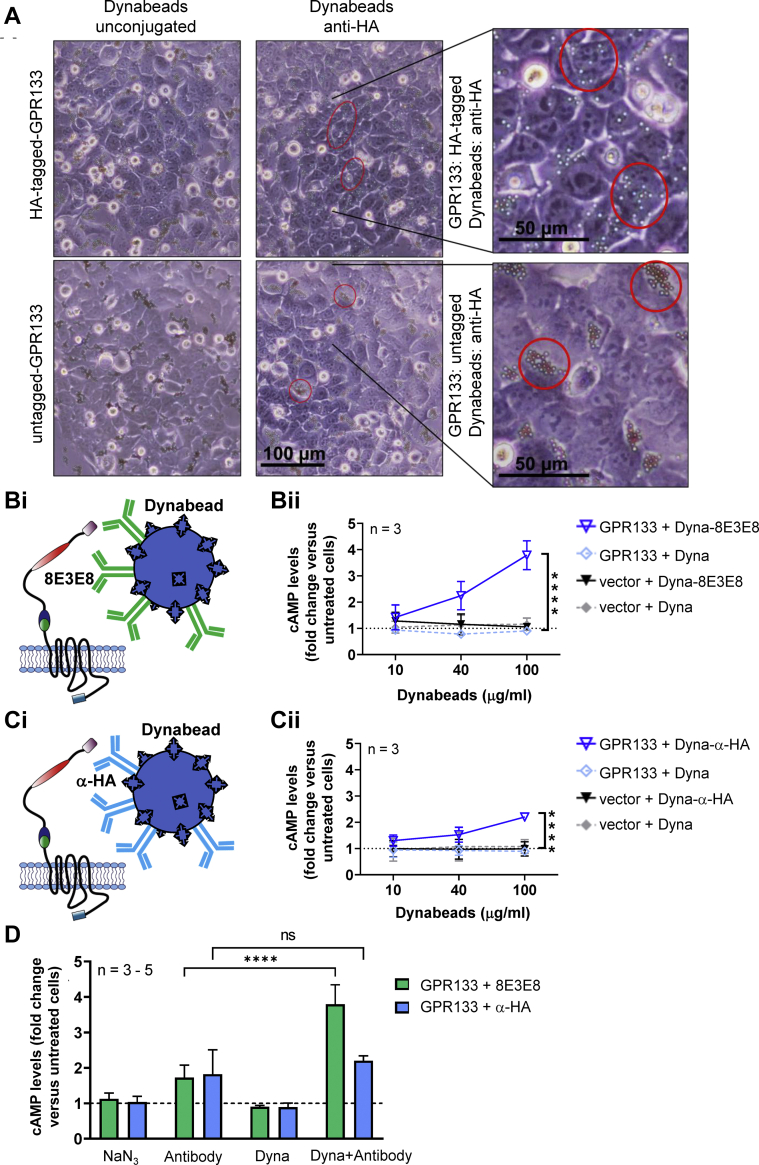
**Stimulation with antibody-conjugated Dynabeads increases cAMP levels in HEK293T cells overexpressing GPR133.***A*, α-HA–conjugated Dynabeads adhere to the plasma membrane of HEK293T cells overexpressing HA-tagged GPR133. Representative micrographs of HEK293T cells treated with Dynabeads. Unconjugated Dynabeads (*top panels*) or α-HA–conjugated Dynabeads (*bottom panels*) were incubated with cells overexpressing untagged GPR133 (*left panel*) or HA-tagged GPR133 (*right panel*). When α-HA–conjugated Dynabeads and HA-tagged GPR133 are present, beads spread out across cell surfaces. In all other conditions, beads cluster between cells. *Red circles* highlight examples of unbound clustered Dynabeads (*left*), as well as cell surface–bound anti-HA–conjugated Dynabeads (*right*). *B* and *C*, HEK293T cells overexpressing a double-tagged GPR133 construct were treated with Dynabeads conjugated to (*Bi*) 8E3E8 or (*Ci*) anti-HA antibodies. Data points represent mean ± SD of three individual experiments (∗∗∗∗*p* < 0.0001). *Bii*, treatment with 8E3E8-conjugated Dynabeads leads to a concentration-dependent increase of cAMP levels compared to the treatment with unconjugated beads (F_(1,12)_ = 82.41, *p* < 0.0001, two-way ANOVA). *Cii*, treatment with α-HA–conjugated Dynabeads significantly increases cAMP levels compared to unconjugated beads (F_(1,12)_ = 56.46, *p* < 0.0001, two-way ANOVA). *D*, comparison of HEK293T cells overexpressing GPR133 following treatment with maximal concentrations of 8E3E8 (1.8 μg/ml) or anti-HA (10 μg/ml) alone and 8E3E8- or α-HA–conjugated Dynabeads (100 μg/ml). NaN_3_ (0.15 mM) or unconjugated Dynabeads (100 μg/ml) were used as control, respectively. Data on the y-axis represent cAMP levels normalized to untreated cells overexpressing GPR133 (∗∗∗∗*p* < 0.0001). Stimulation with 8E3E8-conjugated Dynabeads had a larger effect than stimulation with α-HA–conjugated Dynabeads compared to the treatment with antibodies alone. (F_(1,24)_ = 8.679, *p* = 0.0071, two-way ANOVA; Tukey’s *post hoc* test: GPR133-8E3E8: Antibody *versus* Dyna-Antibody, *p* < 0.0001; GPR133-α-HA: Antibody *versus* Dyna-Antibody, *p* = 0.5044). HA, hemagglutinin; HEK293T, human embryonic kidney 293T.

**Supplementary Figure 6:**
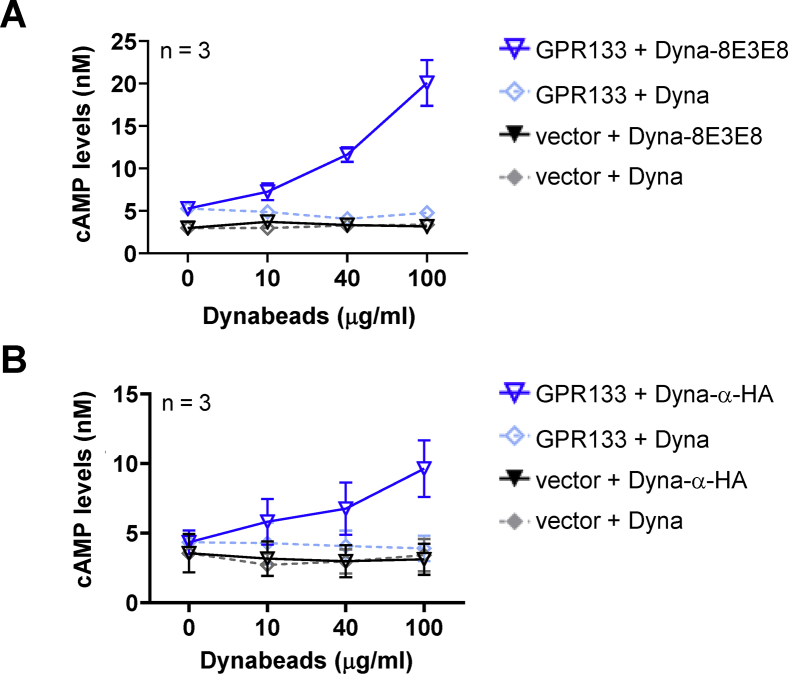


We then tested the hypothesis that increasing the effective concentration or clustering of antibodies at the cell surface would further enhance GPR133 signaling. To accomplish this, we coupled 8E3E8 or the HA-antibody to Dynabeads (Fig. 4) and treated HEK293T cells overexpressing the HA and FLAG-tagged construct of GPR133 (Fig. 4, *A*i and *B*i). We visualized the binding interaction between the antibody-coated beads and HEK293T cells overexpressing GPR133 with light microscopy. Unconjugated Dynabeads served as a control. Dynabeads localized to the plasma membrane of cells only in the condition where they were coated with anti-HA antibody and cells expressed HA-tagged GPR133 (Fig. 4*A*). In the absence of the HA tag in GPR133 or anti-HA antibody coating of the beads, the Dynabeads appear to cluster in spaces between cells (Fig. 4*A*). Furthermore, we tracked the beads’ diffusivity over time by microscopic video capture and found that anti-HA antibody-conjugated Dynabeads had reduced mobility as they bound HA-GPR133 expressing cells, while unconjugated Dynabeads displayed a higher degree of molecular motion, suggesting they remained unbound to cells (Fig. S6). Overall, the imaging findings indicated the specificity of binding of antibody-coated beads to the cell surface.

**Supplementary Figure 7:**
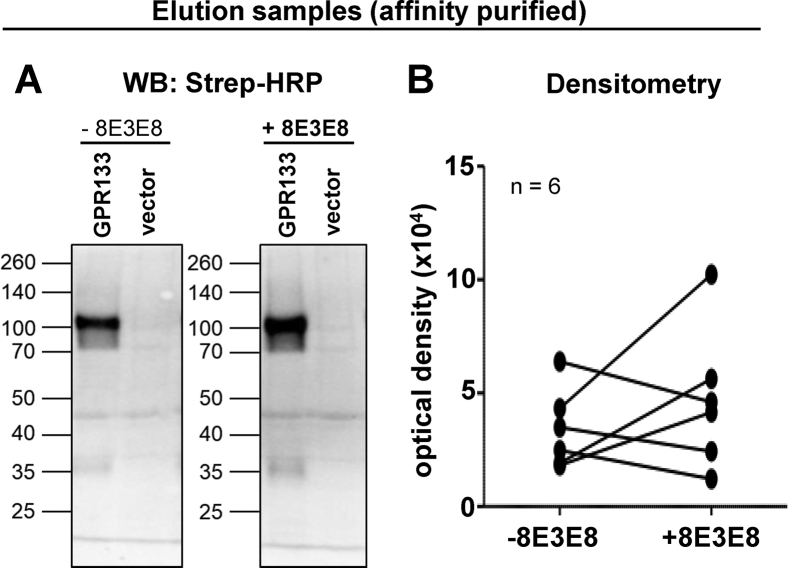


We then treated HEK293T cells overexpressing the HA and FLAG-tagged construct of GPR133 with 8E3E8- or anti-HA–coupled Dynabeads (Fig. 4, *B*i and *C*i; raw cAMP levels in nM are shown in Fig. S7). We used the maximal concentration of antibodies from our dose-response curves (1.8 μg/ml 8E3E8 and 10 μg/ml anti-HA) for coupling to Dynabeads. Intracellular cAMP levels increased significantly after treating the cells with increasing concentrations of 8E3E8-conjugated Dynabeads (Fig. 4*B*ii; F _(1,12)_ = 82.41, *p* < 0.0001, two-way ANOVA) or anti-HA–conjugated Dynabeads (Fig. 4*C*ii; F _(1,12)_ = 56.46, *p* < 0.0001, two-way ANOVA) compared to cells treated with unconjugated Dynabeads. We then compared the magnitude of cAMP increase following the treatment of GPR133-overexpressing HEK293T cells with antibodies alone (8E3E8 or anti-HA) or after conjugation to Dynabeads. We found that 8E3E8-conjugated Dynabeads at 100 μg/ml (3.8-fold when compared to untreated cells) potentiated the agonistic effects of 8E3E8 alone (1.8 μg/ml) more robustly than coupling of anti-HA antibody to Dynabeads (2.2-fold when compared to untreated cells) relative to anti-HA alone (10 μg/ml) (Fig. 4*D*; F _(1,24)_ = 8.679, *p* = 0.0071, two-way ANOVA; Tukey’s *post hoc* test: GPR133-8E3E8: Antibody *versus* Dyna-Antibody, *p* < 0.0001; GPR133-anti-HA: Antibody *versus* Dyna-Antibody, *p* = 0.5044). We theorize that binding of anti-HA to GPR133 might already be saturated at this antibody concentration, which explains the smaller additive effect of anti-HA–coupled Dynabeads compared to 8E3E8-coupled Dynabeads (Fig. 4*D*). The large effect of 8E3E8-coated beads on GPR133 signaling suggests that increased effective concentrations or clustering of antibodies at the cell surface promotes receptor activation. Alternatively, the potentiation of the agonistic effect of antibodies by Dynabeads may be related to the rigid fixation of the antibodies and increased mechanical forces applied to GPR133 through the antibody–receptor interaction.

### GPR133 NTF–antibody complexes in the culture medium

**Figure 5:**
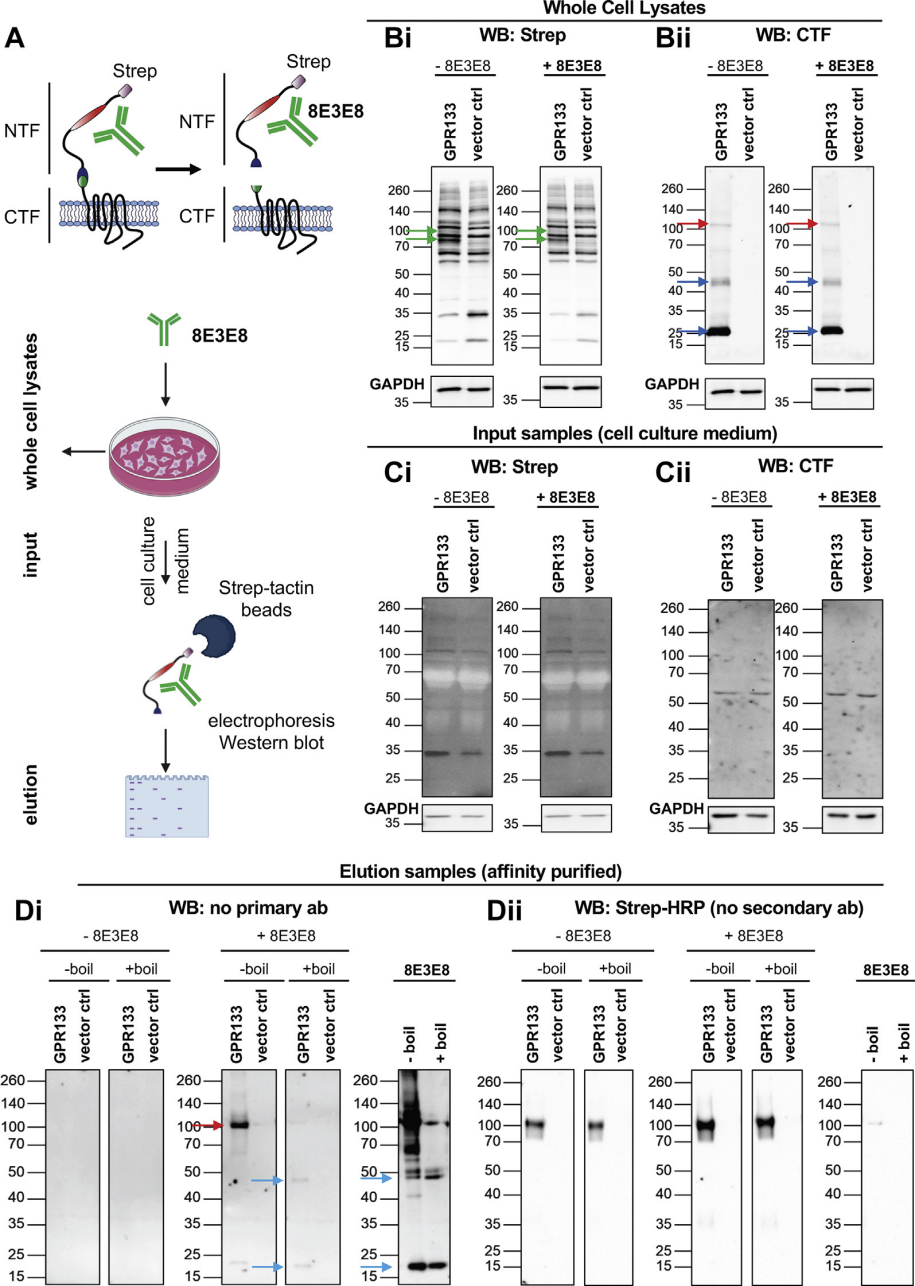
**The 8E3E8 antibody is bound to the GPR133 NTF in the cell culture medium.***A*, experimental outline. GPR133 constructs used for the experiments (n = 6) carried an N-terminal Strep-tag. *B*, representative Western blot of whole cell lysates. *Bi*, an anti-Strep antibody was used to detect the NTF (*green arrow*), and (*Bii*) anti-CTF was used to show the CTF (*blue arrow*) and the uncleaved full-length receptor (*red arrow*). GAPDH served as a loading control. The same membrane was stained with anti-Strep, anti-CTF, and anti-GAPDH antibodies. The GAPDH image was reused for single images of the anti-Strep (Bi) and anti-CTF (*Bii*) blots. *C*, Western blot of input samples, representing the medium of HEK293T cells overexpressing GPR133 with and without treatment with 8E3E8, probed with an anti-Strep antibody (*Ci*) or anti-CTF (*Cii*). The NTF and CTF are not detectable. *D*, Western blot analysis of elution samples and the 8E3E8 antibody itself without the use of a primary antibody. Bands were detected using a secondary antibody only, indicating that 8E3E8 is bound to the GPR133 NTF after affinity purification from the medium (*red arrow*). Boiling of elution samples and 8E3E3 itself resolves heavy and light chains of 8E3E8 (*blue arrows*). *Dii*, Western blot analysis of boiled and unboiled elution samples, using an anti-Strep antibody conjugated to horseradish peroxidase (HRP) that directly detects the GPR133 NTF and obviates the need for a secondary antibody. The elution samples and 8E3E8 were prepared the same way as in Figure 4*D*i. CTF, C-terminal fragment; HEK293T, human embryonic kidney 293T; NTF, N-terminal fragment.

We recently showed that NTF and CTF dissociate from each other at the plasma membrane and observed less GPR133 signaling when dissociation was prevented in a cleavage-deficient GPR133 mutant (24). We therefore hypothesized that the effects of antibodies on GPR133 signaling may be mediated by antibody-induced NTF-CTF dissociation at the plasma membrane, in which case we predicted we would detect NTF–antibody complexes in the cell culture medium (Fig. 5*A*). To test this hypothesis, we transfected HEK293T cells with GPR133 tagged with Twin-Strep-tag at the N-terminus (Fig. 5*A*) and treated them with 1.8 μg/ml 8E3E8, which binds the PTX domain of the N-terminus. Western blot analysis of whole cell lysates using an anti-Strep antibody confirmed that the tagged GPR133 was overexpressed and cleaved as expected (24), with the cleaved immaturely and maturely glycosylated NTF (green arrow) both present (Fig. 5*B*i). The anti-CTF antibody detected the cleaved CTF (blue arrow), as well as small amounts of the uncleaved full-length receptor (red arrow) (Fig. 5*B*ii).

We also collected the culture medium 1 h after antibody treatment. Western blot analysis of the medium with an anti-Strep antibody or an anti-CTF antibody did not show any GPR133-specific bands (Fig. 5*C*i, ii). To test if the 8E3E8 antibody is still bound to the Twin-Strep-tagged NTF after affinity purification from the medium with Strep-Tactin XT-coated magnetic beads, we developed approaches to identifying each component of the hypothesized NTF–8E3E8 complex. First, to identify 8E3E8, we probed Western blots of supernatant eluates after 8E3E8 treatment with an anti-mouse secondary antibody (anti-IgG1) in the absence of a primary antibody (8E3E8 is a mouse monoclonal IgG1 antibody) and detected a band at ∼100 kDa (Fig. 5*D*i, red arrow). To verify that this band represents 8E3E8 that was copurified with the Twin-Strep-tagged NTF, we boiled the elution samples and reran the Western blot, again probing only with the anti-mouse secondary antibody. Boiling revealed the heavy and light chain of the 8E3E8 antibody (Fig. 5*D*i, blue arrows) and produced a banding pattern similar to that of the boiled 8E3E8 antibody (Fig. 5*D*i).

Second, to directly identify NTF, we probed eluates with an anti-Strep antibody (which, like 8E3E8, is a mouse IgG1 antibody) conjugated to horseradish peroxidase (HRP), thus forgoing the need for a secondary antibody. Indeed, we were able to detect a GPR133-specific band at ∼100 kDa representing the maturely glycosylated NTF (Fig. 5*D*ii). These findings suggest that 8E3E8 is bound to the NTF in the culture medium, since both proteins were immunoprecipitated using the Strep tag on the NTF. Because the two components of this complex, NTF and 8E3E8, have near identical electrophoretic mobility on SDS-PAGE, we were able to distinguish them individually using distinct antibodies for immunostaining and boiling prior to electrophoresis. The detection of NTF–8E3E8 complexes in the culture medium raises the possibility that the activating effect of the antibody on GPR133 signaling may be mediated by NTF-CTF dissociation.

**Supplementary Figure 8:**
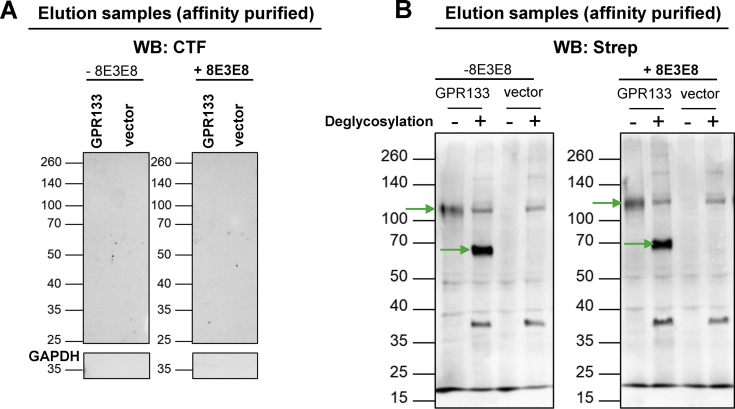


Finally, we analyzed the affinity-purified Strep-tagged NTF of GPR133 in the culture medium by Western blot using the HRP-conjugated anti-Strep antibody (Fig. S8*A*). The densitometric intensity of this protein band was higher in our 8E3E8-treated samples than control untreated samples in 3 of 6 individual experiments, while it decreased in 3 of 6 experiments (Fig. S8*B*). Although this equivocal result did not clarify whether the effect of the antibody is mediated by NTF-CTF dissociation, it is possible that the increase in NTF in half of the trials represents 8E3E8-mediated dissociation of the NTF from the CTF. The fact that this phenomenon was observed in only half of our experiments may be attributable to technical reasons, given that the affinity purification is focused on enrichment of the NTF from the medium through the Twin-Strep-Tag, or otherwise hint at a different mechanism.

**Supplementary Figure 9:**
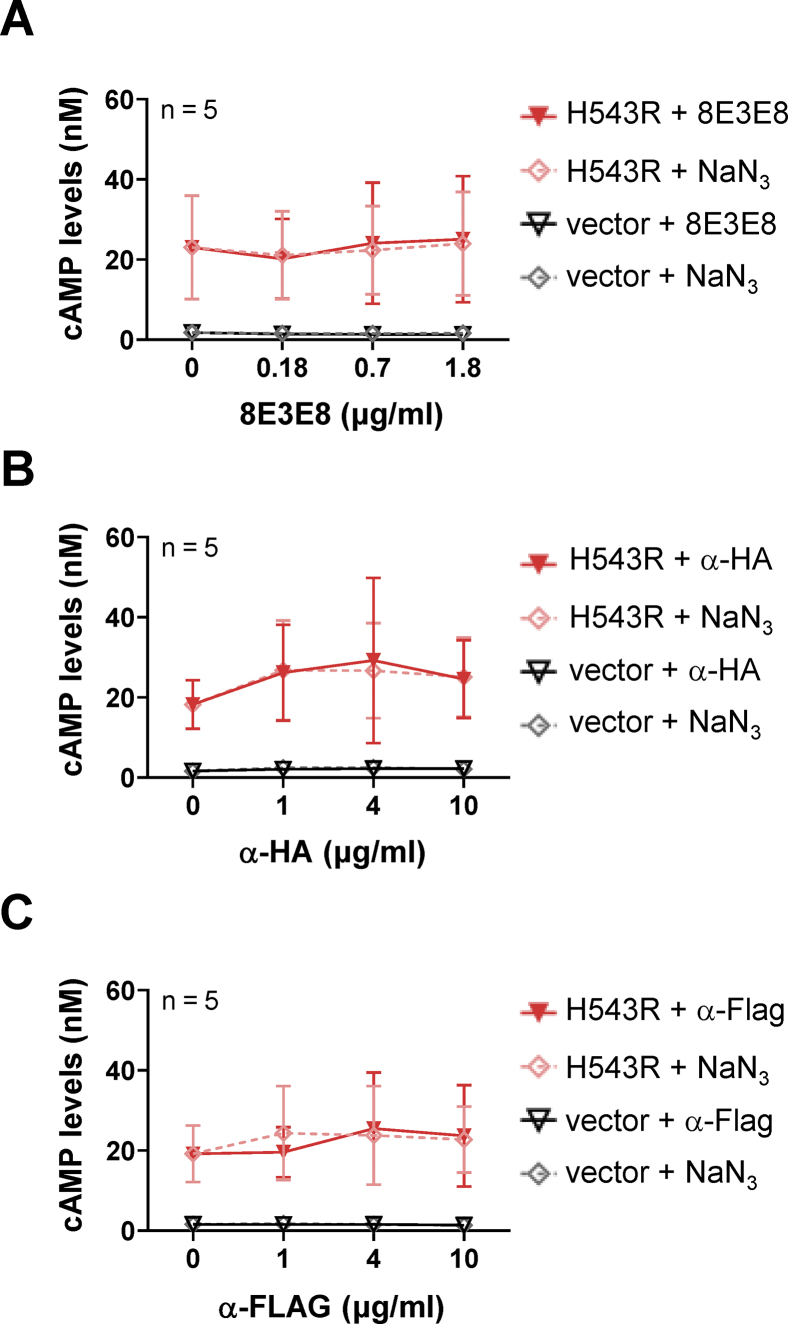


To ensure that the protein band detected at ∼100 kDa in Fig. S8*A* represents the cleaved maturely glycosylated NTF of GPR133 and not the uncleaved form of the full-length receptor, we probed eluates with the anti-CTF antibody and did not detect any GPR133-specific bands before or after treatment with 8E3E8 (Fig. S9*A*). In an additional quality control experiment, elution samples from the culture medium before and after treatment with 8E3E8 were deglycosylated prior to Western blot analysis. Consistent with our prior observations (24), we observed a shift of the apparent molecular weight of the band detected with the anti-Strep antibody from ∼100 kDa to ∼65 kDa (Fig. S9*B*), indicating that the detected band in Figures 4*D*ii and S8*A* indeed represents the maturely glycosylated NTF of GPR133.

### Activation of GPR133 by antibodies depends on receptor cleavage

**Figure 6:**
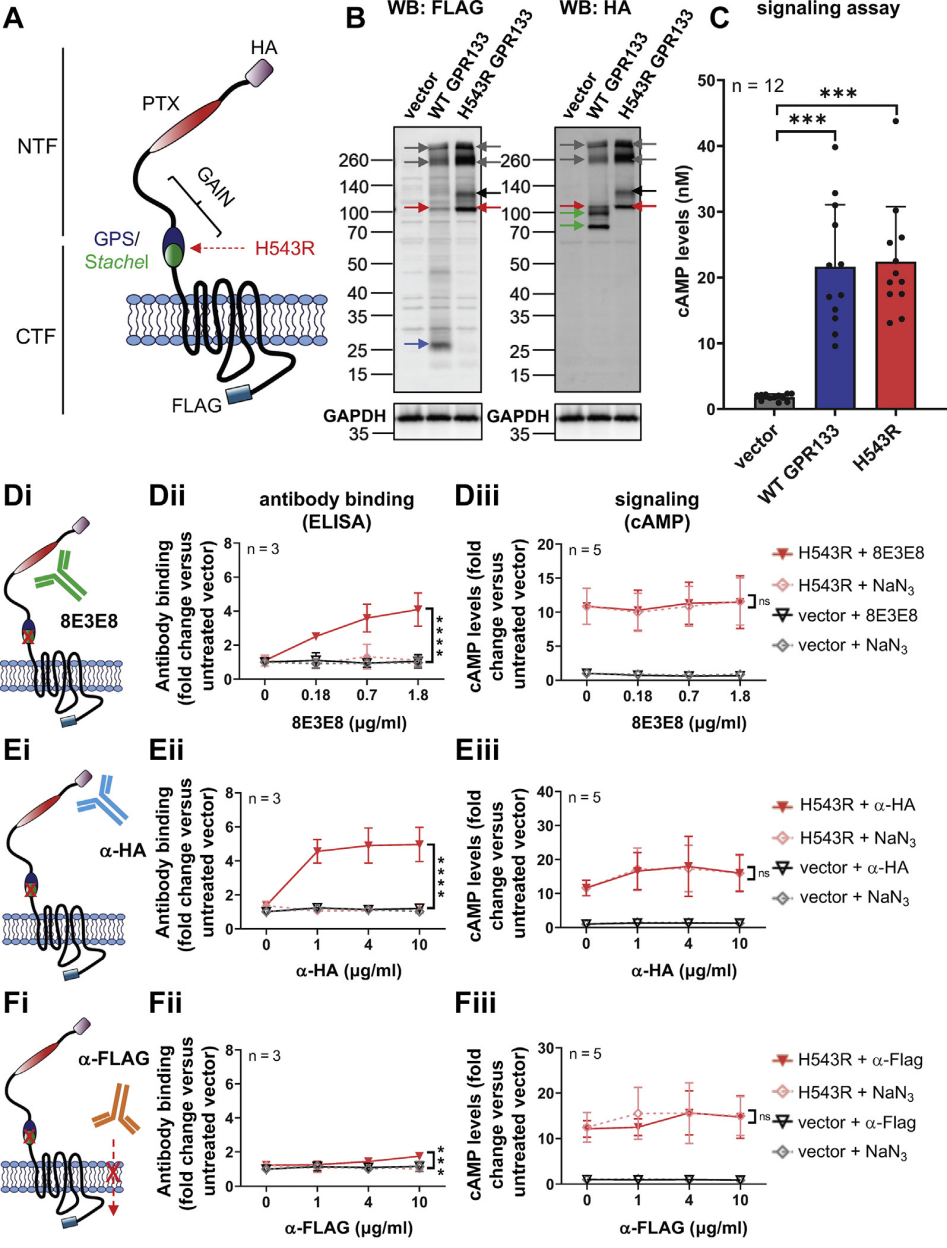
**The cleavage-deficient mutant H543R is not responsive to the antibody stimulus.***A*, the double-tagged GPR133 construct, including an N-terminal HA-tag and a C-terminal FLAG-tag, was mutated at position H543R to prevent receptor cleavage. *B*, overexpression of the WT receptor and the cleavage-deficient mutant H453R is shown by Western blots of whole cell lysates. The same membrane was stained with α-HA antibody (targeting the N-terminal HA-tag), anti-FLAG (targeting the C-terminal FLAG-tag), and anti-GAPDH antibodies. The GAPDH image was reused for single images of the α-HA and anti-FLAG blots. Overexpression of WT GPR133 shows bands representing immaturely glycosylated full-length receptor (*red arrow*), the CTF (*blue arrow*), as well as the immaturely and maturely glycosylated NTF (*green arrow*). Overexpression of H543R shows two bands, representing the maturely (*black arrow*) and immaturely (*red arrow*) glycosylated full-length receptor. The WT receptor and the cleavage-deficient mutant show similar expression levels. *C*, cAMP levels increase after overexpression of H543R. There is no significant difference after overexpression of H543R compared to WT GPR133. Bars represent mean ± SD of four individual experiments (∗∗∗*p* < 0.001). Statistical significance was assessed by one-way ANOVA (F_(2,33)_ = 30.81, *p* < 0.0001, Tukey’s *post hoc* test: vector *versus* WT GPR133, *p* < 0.001; vector *versus* H543R, *p* < 0.001; WT GPR133 *versus* H543R, *p* = 0.9643). *D*–*F*, antibody binding, as assessed by ELISA, and cAMP levels following treatment of HEK293T cells overexpressing H543R with different antibodies. NaN_3_ (0.015 mM, 0.06 mM, 0.15 mM) served as a solvent control. Data points represent mean ± SD of 3 to 5 individual experiments. Statistical significance was assessed by two-way ANOVA (∗∗∗*p* < 0.001; ∗∗∗∗*p* < 0.0001). *D*, 8E3E8 targeting the PTX domain (*Di*) binds to H543R in a dose-dependent manner on ELISA assays (F_(1,16)_ = 54.96, *p* < 0.0001) (*Dii*). Treatment with 8E3E8 does not lead to a concentration-dependent increase of cAMP levels compared to the NaN_3_ treatment (*Diii*; untreated vector = 1.879 ± 0.25 nM cAMP). *E*, the α-HA antibody binds the N-terminal HA tag (*Ei*) in a dose-dependent fashion by ELISA (F_(1,16)_ = 130.1, *p* < 0.0001). *Eii*, treatment with α-HA did not increase cAMP levels compared to the NaN_3_ control (*Eiii*; untreated vector = 1.61 ± 0.25 nM cAMP). *F*, treating HEK293T cells overexpressing H543R with a C-terminal α-FLAG antibody (*Fi*) showed less antibody binding than 8E3E8 or α-HA (F_(1,16)_ = 19.77, *p* = 0.0004) (*Fii*) and no significant changes in cAMP levels (*Fiii*; untreated vector = 1.57 ± 0.24 nM cAMP). CTF, C-terminal fragment; HA, hemagglutinin; HEK293T, human embryonic kidney 293T; NTF, N-terminal fragment; PTX, pentraxin domain.

A prediction of the model that antibody-mediated stimulation of GPR133 signaling relies on NTF-CTF dissociation is that the effect should not occur in uncleavable GPR133 mutants. To test this prediction, we overexpressed a cleavage-deficient point mutant (H543R) GPR133 (24, 25) with an N-terminal HA tag and a C-terminal FLAG tag (Fig. 6*A*) in HEK293T cells. Overexpression of WT GPR133 and the uncleaved receptor was verified by Western blot analysis of whole cell lysates using the anti-HA and anti-FLAG antibodies. Expression of both constructs were similar 48 h after transfection (Fig. 6*B*). Following overexpression of the uncleavable mutant receptor, we detected maturely (black arrow, ∼120 kDa) and immaturely glycosylated (red arrow, ∼110 kDa), full-length H543R GPR133. In contrast to the WT receptor, bands representing the cleaved NTF or CTF were not detectable, indicating lack of cleavage of the mutant receptor. Both antibodies detected bands >260 kDa (gray arrows) following overexpression of WT GPR133 and the uncleaved mutant, presumably representing aggregates of the receptor. HTRF analysis showed significantly higher cAMP levels after overexpression of both WT and H543R GPR133 than those of the empty vector control (F _(2,33)_ = 30.81, *p* < 0.0001, one-way ANOVA; Tukey’s *post hoc* test: vector *versus* WT GPR133, *p* < 0.001; vector *versus* H543R, *p* < 0.001; WT GPR133 *versus* H543R, *p* = 0.9643) (Fig. 6*C*). We then treated HEK293T cells overexpressing H543R with the same antibodies used to stimulate WT GPR133 (8E3E8, anti-HA, and anti-FLAG) (Fig. 6, *D*–*F*), in incrementally increasing concentrations. NaN_3_ served as a solvent control. To verify binding of the antibodies to GPR133, we performed an ELISA under nonpermeabilizing conditions (Fig. 6, *D*ii, *E*ii, *F*ii) and found concentration-dependent binding of the extracellular antibodies (8E3E8: F _(1,16)_ = 54.96, *p* < 0.0001; anti-HA: F _(1,16)_ = 130.1, *p* < 0.0001; two-way ANOVA) to H543R GPR133 (Fig. 6, *D*ii and *E*ii). Similar to our experiments with WT GPR133, optical density after treatment with the intracellular FLAG antibody was significantly increased compared to the NaN_3_ control (Fig. 6*F*ii) (F _(1,16)_ = 19.77, *p* = 0.0004, two-way ANOVA). However, the magnitude of the ELISA signal was much lower than the treatment with 8E3E8 or anti-HA.

**Supplementary Figure 10:**
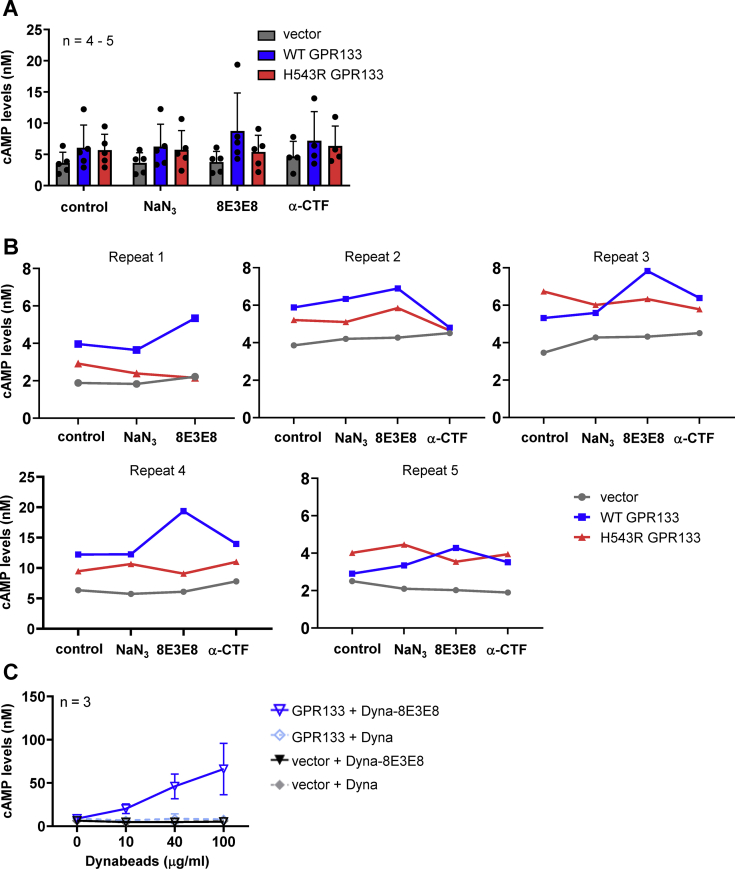


Next, we quantified intracellular cAMP levels following stimulation with the antibodies (Fig. 6, *D*iii, *E*iii, *F*iii; raw cAMP levels in nM are shown in Fig. S10). In contrast to HEK293T cells overexpressing the WT receptor, treatment of cells expressing H543R with 8E3E8 or anti-HA antibodies did not increase cAMP levels compared to the treatment with NaN_3_ (Fig. 6, *D*iii and *E*iii). As a control, we repeated the assay with the anti-FLAG antibody, recognizing the receptor’s C-terminus, which did not activate the mutant receptor (Fig. 6*F*iii). These findings suggest that stimulation of GPR133 signaling by 8E3E8 or anti-HA antibodies is cleavage-dependent.

### Antibody-mediated increase in GPR133 signaling is reproducible in GBM cells

**Figure 7:**
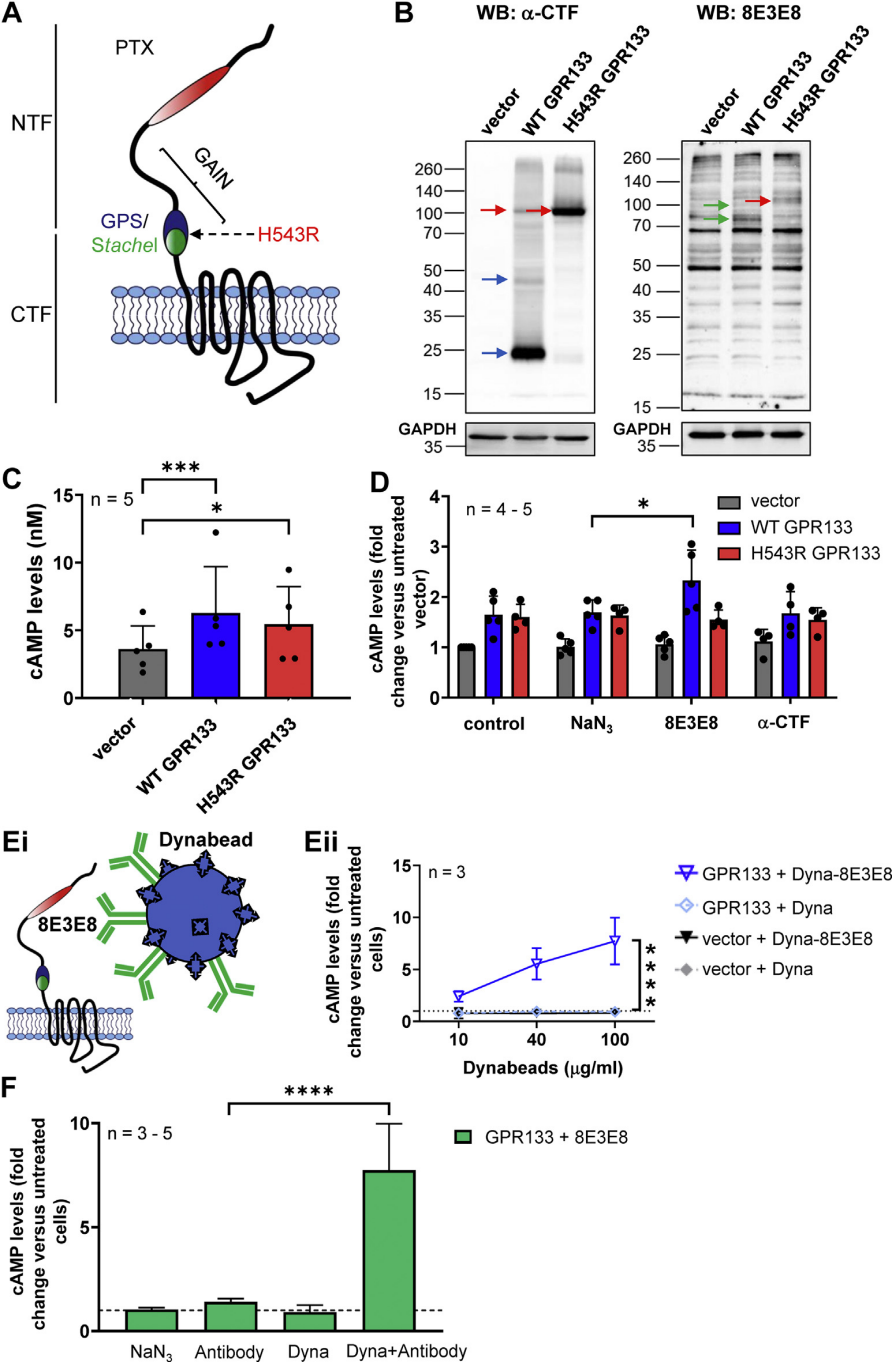
**Antibody stimulation increases cAMP levels in GBM cells overexpressing GPR133.***A*, an untagged version of GPR133 was used for these experiments and the point mutation H543R was introduced to prevent receptor cleavage. *B*, overexpression of GPR133 and the cleavage-deficient mutant H543R in GBML137 is shown by Western blots of whole cell lysates. Upon overexpression of GPR133, the full-length receptor (*red arrow*), the CTF (*blue arrow*), and the NTF (*green arrow*) are detectable. When H543R is overexpressed, only a single band representing the uncleaved full-length receptor (*red arrow*) is detected. *C*, cAMP levels increase significantly after overexpression of GPR133 or H543R compared to the vector control in GBML137 cells (F_(2,12)_ = 14.01, *p* = 0.0007, one way ANOVA, Tukey’s *post hoc* test: vector *versus* GPR133, *p* = 0.0006; vector *versus* H543R, *p* = 0.0113)). Bars represent the mean ± SD of 5 individual experiments (∗*p* < 0.05; ∗∗∗*p* < 0.001). *D*, cAMP levels following treatment of GBML137 cells overexpressing GPR133 with different antibodies. NaN_3_ served as a solvent control. Treatment with 8E3E8 led to a significant increase of cAMP levels in cells overexpressing WT GPR133. The mutant H543R did not respond to the stimulus. Treating GBML137 cells overexpressing WT or H543R GPR133 with a C-terminal antibody did not change cAMP concentrations. Data points are shown as mean ± SD of 4 to five individual experiments (untreated vector = 3.61 ± 0.77 nM cAMP; ∗*p* < 0.05). There was a significant difference between the GPR133 + NaN_3_ and GPR133 + 8E3E8 condition (F_(2,42)_ = 33.66, *p* < 0.0001, two-way ANOVA; Tukey’s *post hoc* test: GPR133 + NaN_3_*versus* GPR133 + 8E3E8, *p* = 0.0091). *Ei*, patient-derived GBM cells (GBML137) overexpressing an untagged GPR133 construct were treated with Dynabeads conjugated to 8E3E8. *Eii*, treatment with 8E3E8-conjugated Dynabeads led to a concentration-dependent increase of cAMP levels in GBML137 cells overexpressing GPR133, compared to the treatment with unconjugated beads. For all experiments, unconjugated Dynabeads served as a control. Data points represent the mean ± SD of 3 individual experiments (∗∗∗∗*p* < 0.0001). Among GPR133-expressing cells, there was a statistically significant difference between treatment with unconjugated Dynabeads and Dynabeads conjugated to 8E3E8 (F_(1,12)_ = 64.00, *p* < 0.0001, two-way ANOVA). *F*, comparison of GBML137 overexpressing GPR133 following treatment with 8E3E8 (1.8 μg/ml) alone and 8E3E8-conjugated Dynabeads (100 μg/ml). NaN_3_ (0.15 mM) or unconjugated Dynabeads (100 μg/ml) were used as controls, respectively. Data on the y-axis represent cAMP levels normalized to untreated GBM cells overexpressing GPR133. Stimulation with 8E3E8-conjugated Dynabeads had a larger effect than stimulation with 8E3E8 alone. (F_(3,12)_ = 1.944, *p* < 0.0001, one-way ANOVA, Tukey’s *post hoc* test: GPR133-8E3E8: Antibody *versus* Dyna-Antibody, *p* < 0.0001). CTF, C-terminal fragment; GBM, glioblastoma; NTF, N-terminal fragment.

Next, we investigated whether our findings with the WT and the cleavage-deficient H543R mutant GPR133 could be reproduced in patient-derived GBM cells. We transfected a patient-derived GBM culture, GBML137, with untagged versions of WT and the cleavage-deficient H543R mutant GPR133, as described in previous studies (24) (Table S1 and Fig. 7*A*). Western Blot analysis of whole cell lysates, using anti-CTF and anti-NTF (8E3E8) antibodies, confirmed that both constructs were expressed in GBM cells in comparable amounts (Fig. 7*B*). In agreement with our previous findings (24), staining with an anti-CTF antibody detected the CTF (blue arrow, ∼25 kDa) and the full-length uncleaved receptor (red arrows, ∼110 kDa). Staining with the 8E3E8 antibody detected bands representing the maturely and immaturely glycosylated NTF (green arrows, ∼95/75 kDa) exclusively in WT GPR133 overexpressing GBM cells. HTRF-based analysis of intracellular cAMP levels showed a significant increase of cAMP after transfection with WT and H543R GPR133 compared to the empty vector (F_(2,12)_ = 14.01, *p* = 0.0007, one way ANOVA; Tukey’s post hoc test: vector *versus* GPR133 *p* = 0.0006; vector *versus* H543R *p* = 0.0113) (Fig. 7*C*). GBM cells overexpressing WT and H543R GPR133 were then treated with the 8E3E8 antibody (1.8 μg/ml) and an antibody targeting the intracellular C-terminus (2 μg/ml anti-CTF). Treatment with 8E3E8 significantly increased cAMP levels relative to the solvent control NaN_3_ in WT GPR133-expressing cells (F_(2,42)_ = 33.66, *p* < 0.0001, two-way ANOVA; Tukey’s *post hoc* test: GPR133 + NaN_3_ *versus* GPR133 + 8E3E8, *p* = 0.0091), but not in cells expressing the uncleaved H543R mutant receptor (Fig. 7*D*; raw cAMP levels in nM are shown in Fig. S11, *A* and *B*). Furthermore, the anti-CTF antibody, which is not expected to permeate the plasma membrane, had no effect on signaling (Fig. 7*D*). In general, we observed lower cAMP levels in GBM cells overexpressing GPR133 than in transfected HEK293T cells, reflecting our previous cAMP measurements in patient-derived GBM cell lines (24). However, the effects of antibody treatment on GPR133 signaling were comparable to the effects we saw in HEK293T cells. We observed a 1.4-fold increase in cAMP levels following treatment of GBML137 overexpressing GPR133 with 1.8 μg/ml 8E3E8 (fold change in cAMP levels relative to untreated empty vector condition: GPR133 + NaN_3_ = 1.69 ± 0.11 *versus* GPR133 + 8E3E8 = 2.32 ± 0.27).

We also tested whether 8E3E8-coated Dynabeads stimulate GPR133 signaling in patient-derived GBM cells (Fig. 7*E*i), similar to their effect in HEK293T cells. Indeed, treatment of GBML137 cells overexpressing GPR133 with 8E3E8-conjugated Dynabeads robustly increased intracellular cAMP levels, compared to cells treated with unconjugated Dynabeads (F _(1,12)_=64.00, *p* < 0.001, two way ANOVA) (Fig. 7*E*ii; raw cAMP levels in nM are shown in Fig. S11*C*). We observed a large boost in signaling when we compared the stimulation with 8E8E8 alone (1.8 μg/ml, 1.4-fold increase, compared to untreated cells) to 8E3E8-conjugated Dynabeads (100 μg/ml, 7.7-fold increase, compared to untreated cells) in GBM cells overexpressing GPR133 (Fig. 7*F*; F_(3,12)_ = 1.944, *p* < 0.0001, one-way ANOVA, Tukey’s *post hoc* test: GPR133-8E3E8: Antibody *versus* Dyna-Antibody, *p* < 0.0001). Overall, these findings indicate that antibody-induced GPR133 activation occurs in multiple cellular contexts, including GBM cells.

## Discussion

**Figure 8:**
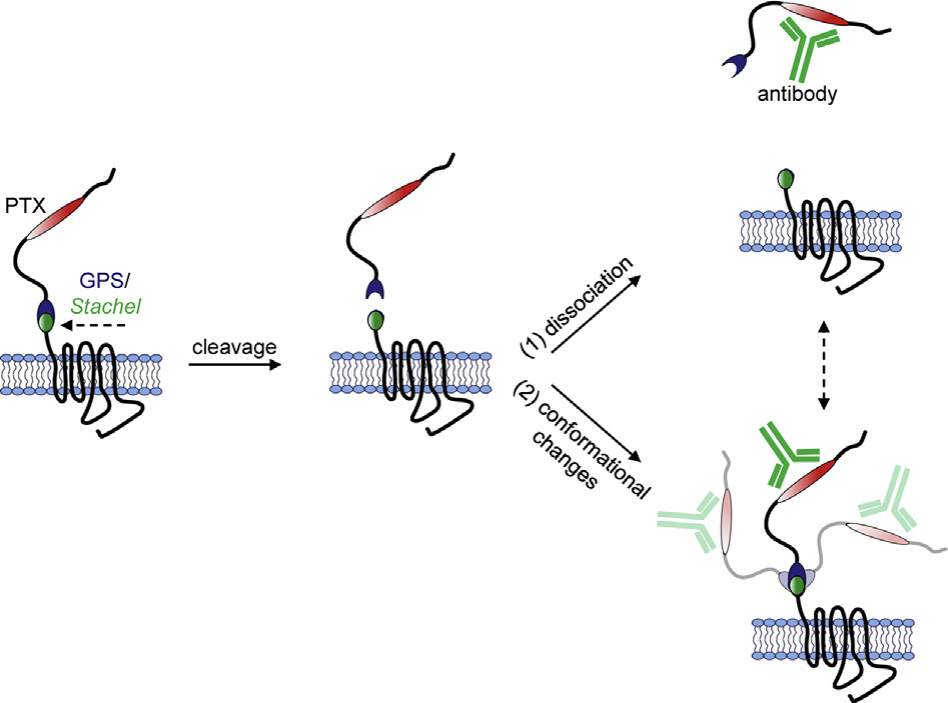
**Proposed mechanisms of GPR133 activation by N-terminal antibody binding.** After autoproteolytic cleavage of GPR133, the NTF and CTF stay noncovalently bound to each other until (1) antibody binding results in increased dissociation of the NTF from the CTF or (2) the antibody causes conformational changes permissive to increased signaling. CTF, C-terminal fragment; NTF, N-terminal fragment.

Our findings provide evidence that the binding of antibodies to the N-terminus of GPR133, proximal to the GAIN domain, results in receptor activation and increased signaling. Following treatment of GPR133-expressing cells with these activating antibodies, we detected antibody–NTF complexes in the culture medium. Furthermore, NTF was enriched in the medium after treatment with the antibody in half of the trials, while it decreased in the other half of our experiments. Finally, the effect depends on the autoproteolytic cleavage of the receptor, because an uncleavable mutant GPR133 (H543R) did not show modulation of its signaling by the same antibodies that activate the WT receptor. These findings raise the possibility that the antibody-induced receptor activation may be mediated by antibody-mediated dissociation of the NTF from the CTF. This mechanism is supported by our previous finding that NTF-CTF dissociation at the plasma membrane correlates with increased receptor signaling (24), as well as the fact that GPR133 deletion mutants lacking the NTF exhibit significantly increased signaling relative to the WT receptor (9). We postulate that this antibody-induced NTF-CTF dissociation may lead to the unveiling of the endogenous tethered agonist immediately distal to the GPS autoproteolysis site, the *Stachel* sequence, and a boost in receptor activation (Fig. 8). This model is supported by the observation that soluble *Stachel* peptide blunts the concentration-dependent agonistic effects of the antibodies.

However, the fact that in half of our trials we do not detect increases in NTF in the culture medium may not just indicate technical variability but may actually suggest an alternative mechanism, not mutually exclusive with the NTF-CTF dissociation model. In this mechanism, antibody binding to the N-terminus may result in conformational changes that impact the GAIN domain and lead to receptor activation. In support of this latter model, Beliu *et al*. reported that the structural flexibility of the GAIN domain contributes to *Stachel* exposure, while simulations suggested that cleavage increases the mobility of the GAIN domain (30). In this scenario, the uncleavable H543R mutant receptor may fail to respond to the antibody because of rigidity imparted by the lack of cleavage and inability to respond to antibody binding with appropriate conformational changes (Fig. 8).

An alternative explanation for the observed increase in NTF in the culture medium that was detected after antibody treatment may be that the antibody helps stabilize spontaneously shed NTF that is released into the medium during the course of the experiment. It was first shown by Araç et al. (8) that recombinant aGPCR NTFs are stable only when the 13th β strand of the GAIN domain is included in the NTF-only expression constructs. This is the portion of the NTF that lies on the C-terminal side of the GPS cut site and is not present in native NTFs that are shed or dissociated from cells. Attempts to produce active, recombinant NTFs that lacked this β strand, akin to the natively shed NTFs, were unsuccessful. These proteins were unstable and rapidly aggregated. Therefore, it must be considered that inclusion of an antibody that can bind to the shed NTF may help stabilize the protein, which could explain its increased abundance in the pull-down experiments.

The specificity of the effect of the 8E3E8 antibody against the PTX domain of the N-terminus was demonstrated in our PTX deletion mutant GPR133. This mutant is autoproteolytically cleaved and is trafficked to the plasma membrane, but its basal level of signaling is reduced relative to WT GPR133. This effect may be due to conformational changes within the extracellular domain, the reduced length of the N-terminus after deleting the PTX domain, or lack of binding to PTX-specific ligands that help activate the receptor. While the PTX deletion mutant GPR133 is not modulated by 8E3E8 treatment, its signaling is still boosted by treatment with the anti-HA antibody when the receptor is HA-tagged, suggesting the underlying mechanisms behind antibody modulation are still active, even when critical domains are missing but cleavage is preserved. The fact that antibody binding to the PTX domain of the N-terminus of GPR133 increases signaling, while PTX deletion has the opposite effect, suggests a crucial function for this particular domain in receptor signaling, possibly through ligand binding. This hypothesis is supported by the recent identification of Plexin Domain-Containing Protein 2 (Plxdc2) as an activating ligand of GPR133, *via* an interaction with the PTX domain (31).

Of particular interest is the observation that coating beads with GPR133-activating antibodies produces an even more pronounced boost in receptor signaling. We theorize that this phenomenon can be explained by different possible mechanisms. First, the coated beads, which in our *in vitro* culture system do not stay suspended in the culture medium but precipitate and make contact with the surface of cells, may significantly increase the effective concentration of antibody at the plasma membrane or promote clustering of the antibodies. Second, the beads, by virtue of their bulk, may represent a rigid surface for attachment of the antibodies, which restricts their mobility and may facilitate dissociation of the antibody-bound NTF from the CTF. In essence, through their constant dynamic motions, the beads may mimic the mechanical force postulated to help pull the NTF off the CTF. Third, the mechanical forces applied onto the receptor by the beads may cause conformational changes which lead to activation. Further experimentation will be required to discriminate among these mechanisms.

Previous studies showed that antibodies and other biologics directed against the extracellular domains of aGPCRs can indeed modulate receptor activation. As an example, treatment of CD97 (ADGRE5) with antibodies targeting the N-terminus outside the GAIN domain results in NTF shedding and formation of complexes containing the antibody and the NTF in the medium of mouse splenocyte cultures (32). Furthermore, a recent study on EMR2 (ADGRE2) showed increased signaling after treatment with an antibody binding the N-terminus (33). These findings are in agreement with ours and support the notion that biologics against the N-terminus of aGPCRs can modulate receptor activation.

Our data indicate that antibody-mediated enhancement of GPR133 signaling depends on the autoproteolytic cleavage of the receptor. This finding raises the possibility that the effect is mediated by NTF-CTF dissociation, or alternatively by antibody binding–dependent conformational changes in the receptor that are enabled by the cleavage but prevented in the uncleavable mutant. Interestingly, there exist examples of other aGPCRs whose modulation by biologics does not depend on this cleavage. For example, treatment of GPR56 (ADGRG1) with monobodies targeting its N-terminus, both inside and outside the GAIN domain, modulates canonical signaling independent of cleavage (34). The discrepancy suggests activation mechanisms may vary among individual aGPCRs. Since both of our signaling-modulating antibodies target N-terminal sequences outside of the GAIN domain of GPR133, it will be interesting to test in the future whether anti-GAIN antibodies elicit effects on NTF-CTF dissociation and signaling and, if yes, the direction of modulation, activating or inhibitory.

In summary, this study provides a paradigm for the use of biologics in modulation of GPR133 signaling. Such biologics can serve as molecular tools toward better understanding receptor activation but can also be used therapeutically in appropriate disease contexts. For example, given our discovery that GPR133 is required for GBM growth, engineering inhibitory anti-GPR133 antibodies would lay the foundation for testing effects on tumor biology (21). Alternatively, given the *de novo* expression of GPR133 in GBM relative to healthy nonneoplastic brain tissue, anti-GPR133 internalizing antibodies could be used to deliver antibody-drug conjugates to GBM cells (21, 29, 35).

## Experimental procedures

### Generation of GPR133 constructs

All GPR133 constructs used in this study are listed in Table S1. HF-GPR133 (HF = N-terminal HA-tag and C-terminal FLAG-tag) was available from previous studies (9, 14). The H543R point mutant from HF-GPR133 was generated by site-directed mutagenesis using the Q5 Site-Directed Mutagenesis Kit (NEB, Cat# E0554S), following the manufacturer’s protocol. The PTX domain deletion construct HA-GPR133 ΔPTX was generated from HA-GPR133 using a two-fragment Gibson reaction. HA-GPR133 does not have a C-terminal FLAG-tag and the coding sequence was cloned into a different backbone than HF-GPR133 (pLVX instead of pcDps, Table S1). Twin-Strep-tagged GPR133 constructs were generated with Gibson assembly, as previously described (24). Primers for mutagenesis and Gibson cloning are listed in Table S2.

### Antibodies

A list of antibodies used in this study is shown in Table S3. For the treatment of GPR133-overexpressing cells, we used commercial anti-HA, anti-FLAG, and anti-CTF antibodies or a mouse monoclonal antibody we raised against the PTX domain of GPR133 (8E3E8) (21, 29). For Western blot analysis, we used anti-HA or 8E3E8 to detect the NTF or the full-length receptor and anti-FLAG or anti-CTF to detect the CTF or the full-length receptor. After treating cells overexpressing a Twin-Strep-tagged construct of GPR133 with 8E3E8, we used an HRP-conjugated anti-Strep antibody to detect the NTF, thus forgoing the need for a secondary antibody.

### Cell culture and transient transfection

HEK293T cells (Takara, Cat# 632180) were cultured in Dulbecco’s modified Eagle’s medium (DMEM, Gibco, Cat# 11965-118), supplemented with sodium pyruvate (Gibco, Cat# 11360070) and 10% fetal bovine serum (FBS; Peak Serum, Cat# PS-FB2) at 37 °C and 5% CO_2_ in humidified room air. Patient-derived GBM cells were cultured in Neurobasal medium (Gibco, Cat# 21103049), supplemented with N2 (Gibco, Cat# 17-502-049), B27 (Gibco, Cat# 12587010), nonessential amino acids (Gibco, Cat# 11140050), and GlutaMax (Gibco, Cat# 35050061) at 37 °C, 5% CO_2_, and 4% O_2_ in humidified air. GBM culture medium was additionally supplemented with 20 ng/ml recombinant basic Fibroblast Growth Factor (R&D, Cat# 233-FB-01M) and 20 ng/ml Epidermal Growth Factor (R&D, Cat# 236-EG-01M) every other day. GBM cultures were established and maintained as previously described (36). In brief, specimens were obtained from patients undergoing surgery for resection of GBM after informed consent (IRB no. 12-01130). Specimens were dissected with surgical blades and enzymatically dissociated with Accutase (Innovative Cell Technologies, Cat# AT104). Glioblastoma cells were grown as attached cultures on cell culture dishes, pretreated with poly-L-ornithine (Sigma, Cat# P4957) and laminin (Thermo Fisher, Cat# 23017015). HEK293T cells were transfected with plasmid DNA using Lipofectamine 2000 (Invitrogen, Cat# 11668-019) and GBM cells were transfected with plasmid DNA using Lipofectamine 2000 Stem reagent (Thermo Fisher, Cat# STEM00008), following the manufacturer’s protocol.

### HTRF-based cAMP assays

Twenty-four hours after transfection, cells were seeded onto 96-well plates, pretreated with poly-L-ornithine and laminin, at a density of 75,000 cells (HEK293T) or 100,000 cells (GBM) per well. Forty-eight (HEK293T cells) to seventy-two (GBM cells) hours after transfection, we added 1 mM 3-isobutyl-1-methylxanthine (IBMX, Sigma-Aldrich, Cat# I7018-100 MG) to the cells and incubated them at 37 °C for 30 to 60 min. Cells were lysed and cAMP concentrations were measured using the cAMP Gs dynamic kit (CisBio, Cat# 62 AM4PEC) on the FlexStation 3 (Molecular Devices) according to the manufacturer’s protocol.

### Treatment of HEK293T or GBM cells with antibodies or peptides

For antibody stimulation experiments, antibodies (listed in Table S3), sodium azide (NaN_3_), which served as a solvent control because all antibodies were stored in solution containing NaN_3_ (0.015 mM, 0.06 mM, 0.15 mM), or antibody-coupled Dynabeads (Thermo Fisher, Cat# 14311D) were added to the cells in cell culture medium 1 h prior to the IBMX treatment. For antibody coupling to Dynabeads, we used 1.8 μg/ml 8E3E8 or 10 μg/ml anti-HA antibody and followed the manufacturer’s protocol. For peptide stimulation experiments, we treated cells with a previously published (9) *Stachel*-derived peptide specifically activating GPR133 (p13; TNFAILMQVVPLE-OH) and an inactive pCTRL (TNAAIAAQVVPLE-OH). Peptides were dissolved as described previously (9, 13). In short, purified peptides were dissolved at 100 mM in 100% DMSO and further diluted into 10 mM stocks (10% DMSO) using a 50 mM, pH 8, Tris buffer. The concentrations of peptide used were 1.0, 0.5, and 0.25 mM. Treatment with appropriate dilutions of DMSO served as a solvent control. Solubility of p13 in aqueous solution was confirmed by measuring absorbance spectra of p13 diluted in PBS at different concentrations using the Nanodrop2000 spectrophotometer (Thermo Scientific) and observing peak absorbance at 195 nm (π-π∗ transition in peptide bond) or 227 and 233 nm (η-π∗ transition in peptide bond); as well as by dynamic light scattering measurements, which were performed as described previously (13). In experiments combining antibody and peptide treatment, cells were first treated with antibody for 1 h at 37 °C, followed by incubation with 0.25 mM p13, 0.25 mM pCTRL, or 0.25% DMSO (dissolved in cell culture medium with 1 mM IBMX) for 30 min at 37 °C. Cells were then lysed and cAMP concentration was measured, as described above.

### Assessment of Dynabead motion

To assess the amount of molecular motion or immobilization of Dynabeads on monolayer cell surfaces, cells were treated with Dynabeads as described above, followed by microscopic video capture for 200 frames over a time-course of 5 s. Videos were analyzed in ImageJ software by thresholding and inverting each frame to allow for specific detection of the Dynabeads as distinct objects of correct size. Detected Dynabeads were then tracked over 200 frames and individual bead motion tracks were plotted superimposed with the same origin to visualize the motion. Mean square displacement was calculated as $MSD\equiv \left({\left(x-x_{0}\right)}^{2}\right)=\frac{1}{N}\overset{N}{\underset{n=1}{\sum }}{\left(x_{n}\left(t\right)-x_{n}\left(0\right)\right)}^{2}$.

### Enzyme-linked immunosorbent assay

HEK293T cells, transfected with the empty vector or GPR133, were seeded onto 96-well plates as described above. Forty-eight hours after transfection (24 h after seeding), antibodies or NaN_3_ were added to the cells in cell culture medium. Cells were washed once with cold HBSS (+Ca^2+^/+Mg^2+^), fixed with 4% paraformaldehyde (Sigma-Aldrich, Cat# P6148) for 20 min at room temperature (RT) and blocked with cell culture medium containing 10% FBS for 1 h at RT. Cells were then incubated with HRP-conjugated secondary antibodies (1:1000, chicken-anti mouse IgG Invitrogen Cat# A15975 or chicken–anti-rabbit IgG Invitrogen Cat# A15987, Table S4) in cell culture medium containing 10% FBS for 1 h at RT. After 3 washes with PBS, cells were incubated with TMB (3,3′, 5,5′-tetramethylbenzidine)-stabilized chromogen (Thermo Fisher, Cat# SB02) for 15 min. The reaction was stopped by adding an equal volume of acidic stop solution (Thermo Fisher, Cat# SS04) and optical density was measured at 450 nm.

### Western blot analysis of whole cell lysates

Cells were washed with PBS and lysed in RIPA buffer (Thermo, Cat#89900) supplemented with Halt protease inhibitor cocktail (Thermo, Cat#78429) and 1% n-dodecyl β-D-maltoside (Thermo, Cat# BN2005). Lysates were incubated for 15 min on ice, sonicated in a water-bath Bioruptor (Diagenode, Cat# UCD-300), and precleared by centrifugation at 15,000*g* for 10 min at 4 °C. Protein concentrations were measured using the DC protein assay kit II (BioRad, Cat# 5000112). Laemmli buffer (BioRad, Cat# 1610747), supplemented with β-mercaptoethanol, was then added and lysates were incubated at 37 °C for 30 min. Twenty microgram of protein were separated by SDS-PAGE and transferred to 0.2 μm nitrocellulose membranes (BioRad, Cat# 1620112). Membranes were blocked in 2% bovine serum albumin (BSA) in TBS-Tween for 1 h at RT and incubated with primary antibodies (listed in Table S3) at 4 °C overnight. Following incubation with Alexa Fluor or HRP-conjugated secondary antibodies (listed in Table S4) for 1 h at RT, images were acquired using the iBrightFL1000 system (Invitrogen). Signals were detected by fluorescence or chemiluminescence (Thermo Scientific, Cat# 34577), depending on the secondary antibody. Densitometric quantification of band intensities was carried out using ImageJ.

### Affinity purification of strep-tagged GPR133

Twin-Strep-tagged GPR133 (Table S1) was affinity-purified from cell culture medium using Strep-Tactin XT-coated magnetic beads (MagStrep "type3" XT Beads, IBA, Cat# 2-4090-002), according to the manufacturer’s protocol. In brief, HEK293T cells were transfected with N-terminally TwinStrep-tagged constructs of WT GPR133, as described above. Twenty-four hours after transfection, cell culture medium was replaced by DMEM supplemented with 2% FBS. Seventy-two hours after transfection, cells were treated with 1.8 μg/ml 8E3E8 antibody for 1 h at 37 °C. Cell culture medium was collected and whole cell lysates were prepared as described above. Culture medium was treated with 10X Buffer W (IBA, Cat# 2-1003-100) and BioLock (IBA, Cat# 2-0205-250) for 15 min on ice and precleared by centrifugation at 15,000*g* for 15 min at 4 °C. The supernatant was incubated with MagStrep "type3" XT Beads overnight at 4 °C. The next day, beads were collected with a magnetic separator and washed 3 times with 1x Buffer W. Proteins were eluted in two consecutive elutions with 1X biotin elution buffer BXT (IBA, Cat# 2-1042-025). After pooling the elutions, Laemmli buffer with β-mercaptoethanol was added and proteins were analyzed by Western blot, as described above.

### Deglycosylation

Whole cell lysates or eluted proteins after affinity purification were treated with Protein Deglycosylation Mix II (NEB, Cat# P6044), according to the manufacturer’s protocol. Deglycosylation was performed under denaturing conditions for 16 h at 37 °C. Samples were stored in Laemmli buffer, supplemented with β-mercaptoethanol, and analyzed by Western blot.

### Immunofluorescent staining

HEK293T cells were transfected and cultured on dishes coated with poly-L-ornithine and laminin, as described above. Cells were fixed with 4% paraformaldehyde for 30 min at RT, followed by block and permeabilization with 10% BSA in PBS supplemented with 0.1% Triton X-100 for 1 h at RT. Cells were then incubated with a primary anti-HA antibody (Sigma, Cat# H3663) in 1% BSA in PBS + 0.1% Triton X-100 at 4 °C overnight. The next day, cells were washed with PBS + 0.1% Triton X-100 and stained with donkey anti-mouse AlexaPlus 488 IgG (H + L) secondary antibody for 1 h at RT. Nuclei were counterstained with 500 ng/ml 4′,6-diamidino-2-phenylindole (DAPI) for 10 min at RT. Microscopy was conducted on a Zeiss Axiovert epifluorescent microscope.

### Statistical analysis

Statistical analysis was performed using GraphPad Prism (version 8.4.3). Population statistics are represented as mean ± SD as indicated. Statistical significance was calculated using either Students *t* test, one-way ANOVA, or two-way ANOVA, with Tukey’s *post hoc* test for multiple comparisons. *p* values <0.05 were considered statistically significant (∗*p* < 0.05; ∗∗*p* < 0.01; ∗∗∗*p* < 0.001; ∗∗∗∗*p* < 0.0001).

## Data availability

All data are contained within the article.

## Supporting information

The article contains supporting information.

## Conflict of interest

D. G. P. and NYU Grossman School of Medicine own an EU and Hong Kong patent titled “Method for treating high-grade gliomas” on the use of GPR133 as a treatment target in glioma. D. G. P. has received consultant fees from Tocagen, Synaptive Medical, Monteris and Robeaute. The authors declare that they have no conflicts of interest with the contents of this article.

## Supporting information

20220318_Stephan et al_Supporting Information

20220318_Stephan et al_Supporting Information
