# Supplementary material for: Activation of the adhesion G protein–coupled receptor GPR133 by antibodies targeting its N-terminus
Source: J Biol Chem. 2022 Apr 18;298(6):101949. doi: 10.1016/j.jbc.2022.101949 (PMC9133650; doi:10.1016/j.jbc.2022.101949)
Supplement: 20220318_Stephan et al_Supporting Information [file mmc1.pdf]

Supporting Information

Supplementary Tables

Supplementary Table 1: GPR133 constructs

name	backbone	tags	cleavage
GPR133 (HF-GPR133)	pcDps	HA (N-terminal)<br>FLAG (C-terminal)	yes
H543R (HF-GPR133 (H543R))	pcDps	HA (N-terminal)<br>FLAG (C-terminal)	no
GPR133 (codon-optimized)	pLVX	-	yes
H543R (codon-optimized)	pLVX	-	no
HA-GPR133	pLVX	HA (N-terminal)	yes
HA-GPR133 ΔPTX	pLVX	HA (N-terminal)	yes
Strep-GPR133	pLVX	Twin-Strep (N-terminal)	yes
Strep-GPR133 (H543R)	pLVX	Twin-Strep (N-terminal)	no

Supplementary Table 2: Primer sequences

primer	sequence
HF-GPR133 (H543R) for	CGCTGCACTCGACTCACCAACTTTG
HF-GPR133 (H543R) rev	GCAGACGGAGTAGGTGAG
ΔPTX Gibson Fragment 1 for	GCATTTACCTGAAAGAGGAACATCCCATCATAACCAACCTGACAG
ΔPTX Gibson Fragment 1 rev	GGTTAGCTCCTTCGGTCCTCC
ΔPTX Gibson Fragment 2 for	GATAACACTGCGGCCAACTTACTTC
ΔPTX Gibson Fragment 2 rev	AGGTTGGTTATGATGGGATGTTCCTCTTTCAGGTAAATGCCTTTG

Supplementary Table 3: Primary antibodies

antibody	target	species	Company	Cat#
anti-HA	HA-tag	mouse	Sigma	H3663-100UL
anti-FLAG	FLAG-tag	rabbit	Sigma	F3165-.2MG
8E3E8	GPR133 NTF	mouse	Genscript	-
anti-CTF	GPR133 CTF	rabbit	Sigma	HPA042395
anti-Strep-HRP	Strep-tag	mouse	IBA	2-1509-001
anti-GAPDH	GAPDH	goat	Invitrogen	PA1-9046

Supplementary Table 4: Secondary antibodies

antibody	detection	species	Company	Cat#
anti-mouse IgG	Alexa Fluor plus 488	donkey	Invitrogen	A32766
anti-rabbit IgG	Alexa Fluor plus 555	donkey	Invitrogen	A32794
anti-goat IgG	Alexa Fluor plus 647	donkey	Invitrogen	A32849
anti-mouse IgG	HRP / chemiluminescence	chicken	Invitrogen	A15975
anti-rabbit IgG	HRP / chemiluminescence	chicken	Invitrogen	A15987

Supplementary Figure legends

Supplementary Figure 1: Increased cAMP levels in HEK293T cells overexpressing GPR133 following antibody stimulation. cAMP levels are shown in nM. Panels **A**, **B** and **C** correspond to **Fig. 1Diii**, **Eiii** and **Fiii**, respectively.

Supplementary Figure 2: Effects of 8E3E8 and the *Stachel* derived peptide on GPR133 signaling. cAMP levels are shown in nM. Panels **A** – **E** correspond to **Fig. 2 A-E.**

Supplementary Figure 3: The *Stachel*-derived peptide (p13) is soluble in aqueous solution. Peptide solubility was tested by comparing absorption spectra (190 nm – 350 nm) of different concentrations of (**A**) p13 and (**B**) the DMSO control. The concentrations of p13 (0.25 mM, 0.5 mM and 1 mM) as well as the DMSO concentrations (0.25%, 0.5% and 1%) correspond to the concentrations that were used for the dose-dependent activation of GPR133 (**Fig. 2**). Peak

absorbance was observed at 195 nm (π-π^* transition in peptide bond). To reduce the background, p13 and DMSO were diluted in PBS. Absorbance peaks of p13 (**A**) in aqueous solution are significantly larger than those of DMSO (**B**) at 195 nm. Furthermore, the absorbance rises with increasing concentrations of p13 in PBS in concentration-dependent fashion, demonstrating the solubility of the peptide.

Supplementary Figure 4: Additional evidence for p13 solubility, including dynamic light scattering measurements. In order to get a strong signal and reduce background, p13 was dissolved at 5 mM in PBS either with or without filtering prior to measurements. (**A**) To confirm that filtration does not remove significant amounts of insoluble p13, we measured absorption spectra (218 nm – 272 nm) before and after filtration of 5 mM p13 in PBS. Background absorbance at > 240 nm was reduced after filtration. Peak absorbance of p13 solution shifted from 233 nm (unfiltered; red curve) to 227 nm (filtered; orange curve), after filtering. Both peaks are within the range of peak absorbance observed for η-π^* transitions within peptide bonds. The small shift in peak absorbance is likely explained by trace contaminant particles within PBS interacting with p13's peptide bonds prior to filtration. These interactions were lost after filtering the sample, causing the shift in absorbance of η-π^* transition of peptide bonds. Most importantly, the amplitude of the absorbance peaks remains essentially the same, suggesting that p13 concentrations are similar before and after filtering. (**B**) Autocorrelation of dynamic light scattering of (**Bi**) PBS or (**Bii**) 5 mM p13 diluted in PBS. (**Bi**) The PBS control shows noise only, suggesting molecules within the solution are too small to detect. (**Bii**) Based on the cumulative fit, the hydrodynamic radius (R_H) of our sample in solution is R_H = 1.3 nm, suggesting that p13 (expected R_H ~ 1 nm) is dissolved in aqueous solution. (**C**) Radii of particles in in the PBS control (**Ci**) or p13 solution (**Cii**), calculated from the regularization fit in (**B**). Both solutions show background molecules with a radius of R_H = 38.320 nm (**Ci**) or R_H = 59.896 nm (**Cii**). A particle with a radius of R_H = 1.120 nm,

representing the expected size of p13, is exclusively detected in the p13 solution (**Cii**), confirming that p13 is soluble in aqueous solution.

Supplementary Figure 5: Accumulation of cAMP in HEK293T cells overexpressing HA-GPR133 and HA-GPR133 ΔPTX following antibody stimulation. The change in cAMP is shown in nM. Panels **A** and **B** correspond to **Fig. 3Ci** and **Cii**, respectively.

Supplementary Figure 6: α-HA-conjugated Dynabeads® are immobilized upon binding to HEK293T cells overexpressing HA-tagged GPR133. Diffusivity of Dynabeads® on cell surfaces was assessed by microscopic video capture and motility tracing in ImageJ. HEK293T cells overexpressing HA-tagged GPR133 were treated with either unconjugated Dynabeads® or α-HA-conjugated Dynabeads®. (**A**) Five second-long brightfield videos consisting of 200 frames were captured under the microscope and analyzed in ImageJ. Each frame was thresholded and inverted, and beads were automatically identified as round objects of the correct size. The motion was tracked through all frames as the displacement of the center of each detected object, creating a motion path for each Dynabead®. (**Bi, Biii**) The motion paths of all Dynabeads® within each condition were superimposed with the same origin, and (**Bii, Biv**) the mean square displacement of all beads within each experimental condition was plotted as a function of time (frames). α-HA-conjugated Dynabeads® were relatively immobile on HA-GPR133-expressing HEK293T cells when compared to unconjugated Dynabeads® (Diffusion coefficient x1,000 of 1.07 and 9.73, respectively).

Supplementary Figure 7: Increased cAMP levels in HEK293T cells overexpressing GPR133 following stimulation with antibody-coupled Dynabeads. cAMP levels are shown in nM. Panels **A** and **B** correspond to Fig. **4Bii** and **Cii**, respectively.

Supplementary Figure 8: Densitometric analysis of the GPR133 NTF in eluates following antibody treatment. (**A**) Elution of culture medium samples with and without treatment with

8E3E8 after Streptactin® purification of TwinStrep-tagged GPR133. Using an anti-Strep antibody, a protein band representing the NTF of GPR133 at ~100 kDa is detected (one representative western blot out of six individual experiments is shown). (**B**) Densitometry analysis of the elution band shown in (**A**). Data points represent six individual experiments. The intensity of the band representing the NTF increased in 3 out of 6 individual experiments.

Supplementary Figure 9: Quality controls in eluates. HEK293T cells overexpressing GPR133 were treated with the 8E3E8 antibody, followed by Streptactin® purification of the supernatant, and analysis of eluates by Western blot. (**A**) Probing with anti-CTF antibody shows no signal. (**B**) Deglycosylation of elution samples following treatment with 8E3E8. The blot was stained with the anti-Strep antibody. Green arrows point to the NTF.

Supplementary Figure 10: Unchanged cAMP levels in HEK293T cells overexpressing GPR133 H543R following antibody stimulation. cAMP levels are shown in nM. Panels **A**, **B** and **C** correspond to Fig. **6Diii**, **Eiii** and **Fiii**, respectively.

Supplementary Figure 11: Increased cAMP levels in GBM cells overexpressing GPR133 following stimulation with antibodies or antibody-coupled Dynabeads. Panels **A** and **B** correspond to **Fig. 7D. Panel C** corresponds to **Fig.7Eii**. (**A, B**) The change in cAMP following treatment with antibodies is shown in nM. (**A**) Bars represent the mean ± SD of 4-5 individual experiments. (**B**) Data points for each individual experiment. Antibody treatments of GBM cells overexpressing the empty vector, WT GPR133 or GPR133 H543R were performed in paired fashion. (**C**) The increase in cAMP following treatment with antibody-coupled Dynabeads® is shown in nM.
