# Supplementary material for: Activation of the adhesion G protein–coupled receptor GPR133 by antibodies targeting its N-terminus
Source: J Biol Chem. 2022 Apr 18;298(6):101949. doi: 10.1016/j.jbc.2022.101949 (PMC9133650; doi:10.1016/j.jbc.2022.101949)
Supplement: 20220318_Stephan et al_Supporting Information [file mmc2.pdf]

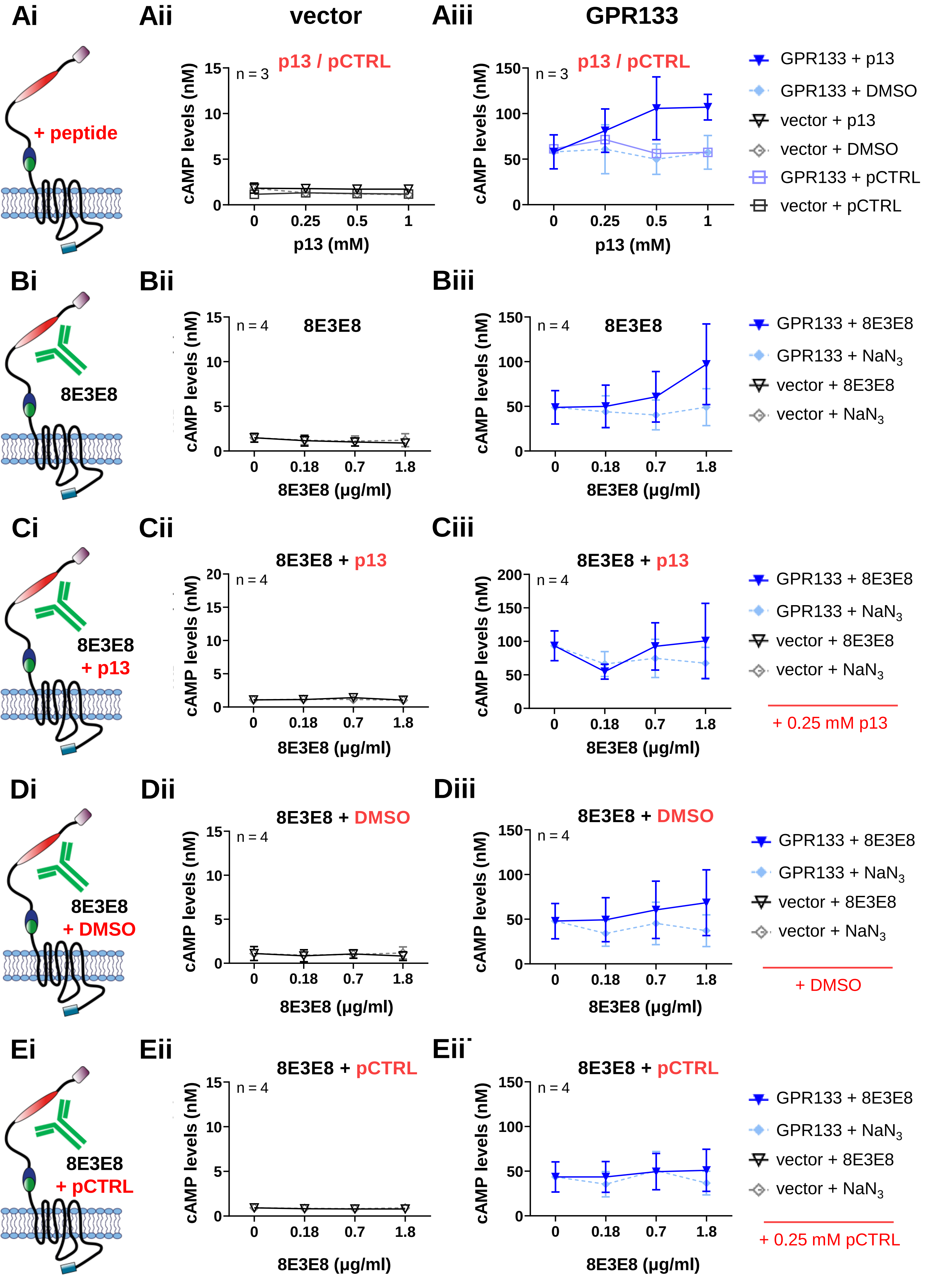

Ai
Aii
vector
Aiii
GPR133
+ peptide
n = 3
p13 / pCTRL
cAMP levels (nM)
p13 (mM)
GPR133 + p13
GPR133 + DMSO
vector + p13
vector + DMSO
GPR133 + pCTRL
vector + pCTRL
Bi
Bii
Biii
8E3E8
n = 4
8E3E8 (µg/ml)
GPR133 + 8E3E8
GPR133 + $NaN_3$
vector + 8E3E8
vector + $NaN_3$
Ci
Cii
Ciii
8E3E8
+ p13
8E3E8 + p13
+ 0.25 mM p13
Di
Dii
Diii
8E3E8
+ DMSO
8E3E8 + DMSO
+ DMSO
Ei
Eii
Eiii
8E3E8
+ pCTRL
8E3E8 + pCTRL
+ 0.25 mM pCTRL
